# Patterning of Artificial Cell Populations Via Laser Induced Forward Transfer for Controlled and Localized Cargo Uptake and Release

**DOI:** 10.1002/smll.76083

**Published:** 2026-09-30

**Authors:** Yiğitcan Sümbelli, Núria Ginés Rodriguez, Lars J.M.M. Paffen, Arjan Hazegh Nikroo, Riccardo Levato, Jan C.M. van Hest, Jos Malda

**Affiliations:** 1 Department of Biomedical Engineering, Institute for Complex Molecular Systems, Eindhoven University of Technology, Eindhoven, The Netherlands; 2 Department of Orthopedics, University Medical Center Utrecht, Utrecht University, Utrecht, The Netherlands; 3 Regenerative Medicine Center Utrecht, Utrecht, The Netherlands; 4 Department of Clinical Sciences, Faculty of Veterinary Medicine, Utrecht University, Utrecht, The Netherlands

**Keywords:** synthetic cell, coacervate, biofabrication, laser induced forward transfer, micropatterning, spatial organization, cell adhesion

## Abstract

Complex coacervate-based synthetic cells are widely used to mimic biologically relevant cellular processes, most often from the single-cell perspective. However, in nature, multicellular organization is a crucial driver of function. Synthetic cell research, therefore, requires fabrication methods that allow control over the spatial organization of synthetic cells to achieve translational potential. Here, we developed an approach to position polymer-stabilized complex coacervates within biopolymer matrices in an adherent manner. To accurately deliver and pattern the coacervates onto tissue culture substrates, we investigated laser-induced forward transfer (LIFT) 3D printing and screened printing parameters, including laser energy and the number of stacked layers, to optimize the printability of the complex coacervate-adhered biomaterial ink. We demonstrated precise spatial control over coacervate localization at the population level by co-patterning multiple coacervate populations into a biomaterial matrix. Lastly, we showed that both the adhered complex-coacervation method and LIFT printing process preserve basic biologically relevant functionality, as demonstrated by protein cargo uptake into and release from the patterned synthetic cells.

## 1 Introduction

Coacervates are spherical droplets formed via liquid–liquid phase separation (LLPS), featuring concentrated high-density polyelectrolyte phases within a dilute phase [1]. They can be found within natural cells as native elements of structural complexity, creating molecularly crowded, localized regions specialized for distinct functions (i.e., membraneless organelles) [2]. These systems, when mimicked in vitro, are often transient and prone to coalescence and instability. To address this issue, their interface can be stabilized, for example, with a membrane-forming polymer component, giving coacervates a longer shelf life and greater resilience to manipulation, printing, and impact [3]. As of now, coacervates have gained interest as delivery carriers for pharmaceuticals and other bioactive molecules, and are commonly used in synthetic cell research due to their ease of fabrication, transmembrane delivery capabilities, ability to sequester molecules in compartments, and capacity to achieve spatiotemporal control over basic cellular functionality in vitro [1, 4]. By increasing the structural robustness of coacervates, their application domain as synthetic cells has been expanded to the area of tissue engineering [5–7].

Tissue engineering focuses on the generation of novel biomaterials with cells that mimic the functional development of human tissue. One way to increase complexity and enhance biological function in engineered tissues is to incorporate microarchitectures. Such microarchitectures include the precise placement of materials and cells, the generation of gradients, such as oxygen tension, and the local delivery of cytokines and small molecules [8–13]. The field of biofabrication encompasses technologies that enable the precise localization and dispensing of biomaterials and cells in complex 3D geometries, including micropatterning applications. These technologies are mostly focused on droplet generators, such as nozzle-based (e.g., inkjet printing) or laser-based (e.g., laser-induced forward transfer (LIFT)) approaches [14, 15]. Common issues with nozzle-based technologies, though, include nozzle clogging, limited range of printable materials, and limitations in cell concentration in the bioink, which greatly hinder their widespread application for micropatterning [16]. Laser-based technologies have been developed as contactless, direct-writing droplet generators that bypass the limitations associated with nozzle-based printers. LIFT uses a nano-pulsed laser to propel micrometer-sized droplets towards a receiver. The laser pulse is directed towards a sacrificial metalized layer (dynamic-release layer), which generates a high-pressure bubble that ejects the biomaterial film deposited on top toward the collector plate. Therefore, the laser pulse does not directly interact with the donor biomaterial [16, 17]. Due to its contactless printing mechanism, LIFT printing has very low material limitations. Printability is defined as the correlation between laser energy, bioink viscosity, and surface tension, with reported printing of a wide range of materials from metals and semiconductors as well as bacteria [18], to biomaterial inks [19], single cells, cell suspensions [20, 21], spheroids [22], and organoids. LIFT generates droplets between 50 and 200 μm in diameter in a fast and reproducible manner [19, 23–26]. In addition, as a noncontact, nozzle-free printing technique, LIFT mitigates cross-contamination and eliminates clogging, while avoiding orifice shear-induced stress. We hypothesize that the use of LIFT to micropattern complex artificial cell systems will enable to locally deliver highly stable synthetic microcarriers loaded with bioactive molecules with high efficiency and resolution. An approach that, so far, and to the best of our knowledge, remains underexplored.

Here, we employed LIFT to position synthetic cell populations within biomaterial matrices with high spatial control. This approach offers a single-step protocol for producing a biomaterial ink in which adhered synthetic cells are embedded based on complex coacervates. We first optimized the complex coacervate LLPS composition to determine the most suitable polypeptide charge ratio for the LIFT process. We demonstrated that altering the order of component addition and the charge ratios affects the size and structural integrity of the adhered complex coacervates, which in turn directly influences their printability with LIFT.

We then investigated the optimal LIFT printing parameters via iterations in laser energy and the number of accumulated layers to maximize the number of deposited coacervates per printed droplet without compromising print accuracy. With optimized printing conditions, we achieved co-patterning of two distinct coacervate populations in mutually exclusive, minimally overlapping designs. Lastly, we explored the use of micropatterning to spatiotemporally control biologically relevant functionality through the localized uptake and release of protein cargo molecules in coacervate populations. Overall, this study describes, for the first time, the successful laser-based patterning of coacervate-based synthetic cells into complex architectures and demonstrates their biomimetic functionality after printing. The precise, spatiotemporal control of structurally stable, functional synthetic cells that can be achieved offers potential in both synthetic biology and biofabrication.

## 2 Results and Discussion

### 2.1 Preparation of Biomaterial Ink Containing Adhered Complex Coacervate-Based Artificial Cells

Commonly used artificial cell-embedded biomaterial ink preparation protocols follow a methodology in which the artificial cells and the ink material are combined as separate entities [27, 28]. This method keeps artificial cells suspended and structurally stable in the matrix, primarily due to the ink’s viscosity and high hydration degrees. However, when substantially low amounts of ink (less than 1 microliter) are used to engineer a 3D structure, rapid drying rates can cause structural disruption of the artificial cells. This can be addressed by introducing rapid crosslinking mechanisms upon printing and increasing the interaction between the artificial cells and the biomaterial ink. We therefore developed a coacervation protocol within a viscoelastic biomaterial ink environment to create in situ complex coacervate-based artificial cells that adhere to the matrix. Furthermore, to introduce rapid crosslinking upon printing, we employed fibrinogen as a biomaterial ink because of its crosslinking mechanism into fibrin upon interaction with a thrombin-containing matrix.

**Figure 1:**
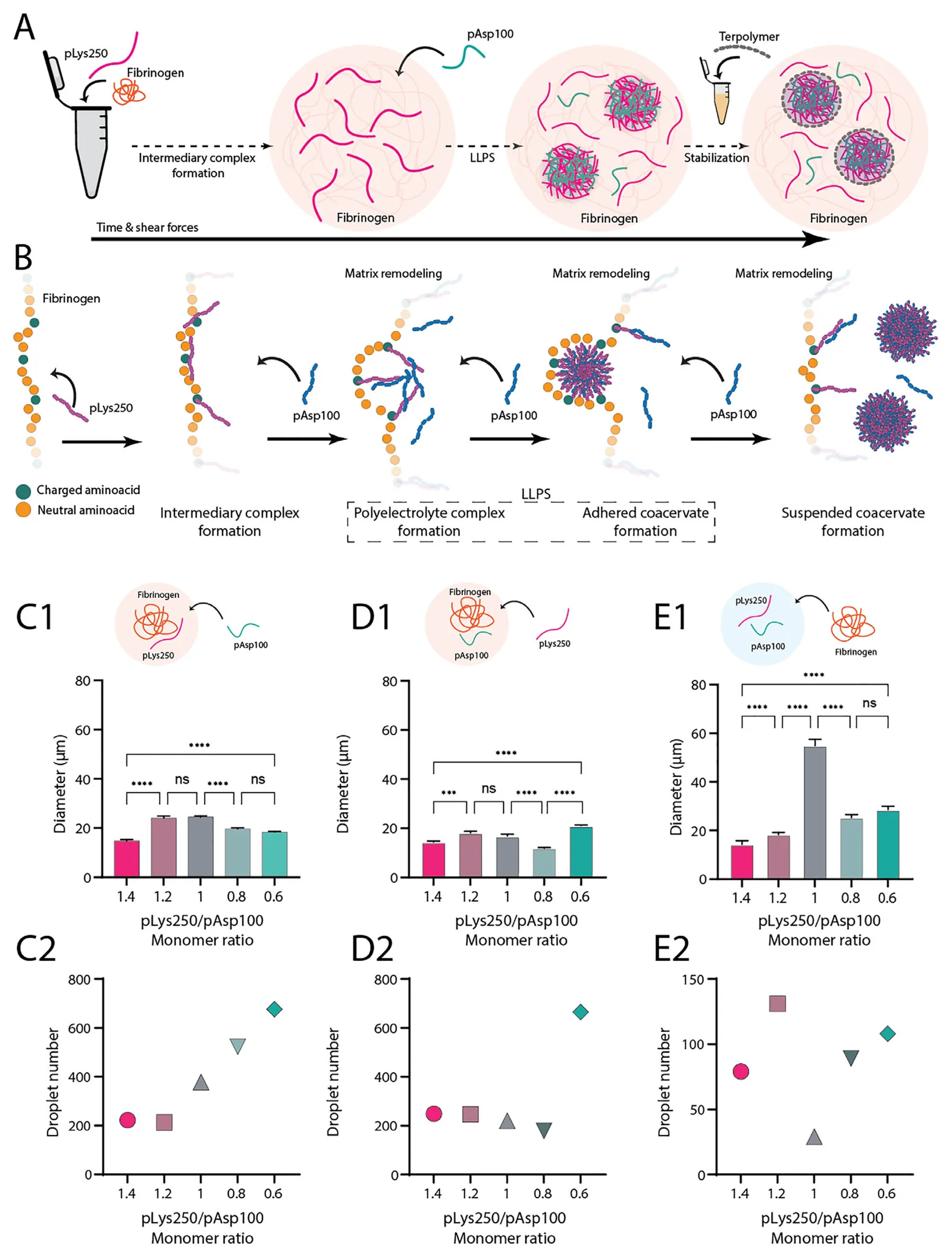
Schematic for the preparation process of biomaterial ink with adhered complex coacervate-based artificial cells. (A) Fibrinogen, the biomaterial ink matrix, and poly-L-lysine (250 monomeric units) (pLys250) form an intermediary complex, which is used to induce liquid–liquid phase separation (LLPS) upon the addition of poly-(L-aspartic acid) (100 monomeric units) (pAsp100). The coacervate droplets are stabilized by the addition of a terpolymer membrane-forming component. (B) The schematics illustrate the adhered complex coacervation process. First, intermediary complex formation is attained upon addition of pLys250 to the matrix material fibrinogen. Subsequently, upon addition of the oppositely charged polypeptide pAsp100 LLPS is induced in proximity to fibrinogen. While complex coacervate droplets grow along the matrix, at a critical stoichiometry between the matrix and the polypeptides, they begin to detach from it. Graphs showing the changes in coacervate droplet size across different monomer ratios when (C1) fibrinogen was premixed with pLys250, (D1) fibrinogen was premixed with pAsp100, and (E1) pLys250 was premixed with pAsp100 prior to fibrinogen addition. Graphs showing the changes in coacervate droplet number across different monomer ratios when (C2) fibrinogen was premixed with pLys250, (D2) fibrinogen was premixed with pAsp100, and (E2) pLys250 was premixed with pAsp100 prior to fibrinogen addition. Graphs show mean ± SEM.

The complex coacervates were composed of poly-L-lysine (250 monomeric units) (pLys250) and poly-(L-aspartic acid) (100 monomeric units) (pAsp100). They were prepared in a viscoelastic fibrinogen environment under constant shear. The formed complex coacervate droplets were then stabilized by the addition of a membrane-forming terpolymer, poly(ethylene glycol)-poly-(caprolactone-gradient-trimethylene carbonate)-poly-(glutamic acid) (PEG-p(CL-g-TMC)-pGlu (Figure 1A). During the adhered complex coacervation, we initially formed an intermediary complex by mixing fibrinogen and pLys250, to ensure the complex coacervation proceeded bound to the fibrinogen matrix. This complex was formed via electrostatic interactions between the negatively charged regions of fibrinogen and the positively charged polypeptide. When we introduced the negatively charged polypeptide pAsp100, the oppositely charged polypeptides started to undergo LLPS. The gradual increase in pAsp100 concentration led to the formation of fibrinogen-adhered complex coacervates up to a monomer ratio of pLys250 and pAsp100 of 1. Upon further increase in [pAsp100] the electrostatic interactions between pLys250 and pAsp100 became dominant, and the coacervates were released from the fibrinogen matrix (Figure 1B).

The complex coacervate adhered biomaterial ink preparation method resulted in variations in the size and number of the formed complex coacervates. Traditionally, nano-/microcarriers are mixed with the matrix component after they are stabilized in a liquid environment (Figure S1). We therefore optimized the preparation protocol by varying the order of component additions to assess changes in the structural properties of the complex coacervate (Figure S2A). Across all tested order-of-addition methods, when the monomer ratio was higher than 1 (i.e., an excess of pLys250 relative to pAsp100), intermediary complex formation was more pronounced, resulting in irregular, precipitate-like structures with fewer coacervate droplets. At these conditions, the system lacked sufficient amounts of pAsp100 to fully reorganize towards complex coacervation. More interestingly, the concentration of pLys250 within the coacervates was lower, suggesting the presence of a pLys250-fibrinogen complex in the medium (Figure S2B,C). A similar trend was observed when the fibrinogen was added to the preformed droplets, indicating strong pLys250-fibrinogen interactions at high [pLys250] conditions (Figure S2D).

When LLPS was initiated on a fibrinogen-pLys250 intermediary complex, coacervate formation was observed at every point on the pLys250/pAsp100 monomer ratio scale, meaning even lower amounts of pAsp100 were enough to form coacervate droplets. In this order of addition strategy, the droplet size varied with the monomer ratio (Figure 1C1). More interestingly, the droplet number increased with the increasing pAsp100 amount (Figure 1C2). Contrarily, when pLys250 was added to the fibrinogen-pAsp100 mixture, in addition to the varied droplet sizes across monomer ratios (Figure 1D1), the number of droplets remained relatively stable compared with the previous preparation strategy (Figure 1D2). Instead of forming an intermediary complex beforehand, adding fibrinogen during LLPS resulted in complex formation only at higher pLys250/pAsp100 monomer ratios. The differences in size (Figure 1E1) and droplet number (Figure 1E2) indicated stochastic interactions between the polymer matrix and the preformed droplets. Taken together, observations of pLys250-fibrinogen complexes only at monomer ratios greater than 1 across all three addition order methods suggest the electrostatic nature of the matrix-polypeptide interactions. Fibrinogen’s isoelectric point is approximately 5.8, which makes it carry a net negative charge at conditions where pH is 7.2. This explains the presence of intermediary complexes when the concentration of the positively charged component was high.

To further confirm the interactions between fibrinogen and coacervate droplets, we centrifuged the samples at low centrifugal force (200 × g) and resuspended the precipitates after removing the supernatant. While the decrease in droplet number after resuspension indicated loss of unadhered coacervates in the samples in which the LLPS was induced on the fibrinogen-pLys250 intermediary complexes, the remaining number suggested the presence of matrix-adhered droplets, especially in the conditions where the monomer ratio was greater than 1 (Figure S3A). Interestingly, the decrease in the droplet number was greater in the condition where the LLPS was induced on the fibrinogen-pAsp100 complex (Figure S3B). This behavior suggested that, despite the presence of the intermediary complexes, the coacervates formed were not matrix-adherent. This result further highlights the electrostatic interactions between fibrinogen and polypeptides, which did not initially occur under the latter order of addition protocol. When the pLys250 and fibrinogen interacted prior to the LLPS, the coacervate formation occurred primarily adhered to the polymer matrix, resulting in a lesser decrease in the droplet number after separation of the supernatant from the precipitated matrix. In addition, we cannot rule out the possibility of the loss of structural stability during the separation process. Yet the presence of coacervates suggested that matrix adhesion could maintain structural protection against mechanical disturbances. This was more prominent when fibrinogen was added to the pLys250-pAsp100 mixture, where droplet numbers were greatly reduced compared to the intermediary complex formation pathways.

These results ultimately confirmed the importance of the order of addition, along with the strength of electrostatic interactions, in driving LLPS [29]. When multiple components carrying the same charge participate in the LLPS process, the initial interactions trigger preliminary complexation between oppositely charged components. It has been suggested that the strength of electrostatic interactions further governs complex coacervation [30]. When both fibrinogen and pAsp100 compete to bind pLys250, the stochasticity of their interactions hinders complex coacervation, consistent with the non-linear changes in coacervate size with changes in monomer ratio. As pAsp100 interacts more strongly with pLys250 than fibrinogen, it ultimately pulls the polycationic species away from the intermediary complexes, as suggested in the consistent increase in the coacervate number.

To further investigate the intermediary complex formation mechanism, we first reduced the polypeptide concentrations in the system in the absence of the fibrinogen matrix. This led to larger coacervate droplets than those prepared at higher polypeptide concentrations (Figures S1–S4). When LLPS was initiated from the fibrinogen-pLys250 intermediary complexes, the resulting coacervates were, however, similar in size to those observed at higher polypeptide concentrations (Figure S5). When LLPS was initiated from the fibrinogen-pAsp100 mixture, no observable coacervate droplets were formed unless the pLys250/pAsp100 monomer ratio was at its lowest. At this condition, the coacervate droplets were very small. This behavior can be explained by an insufficient total polypeptide concentration to drive complex coacervation, while both fibrinogen and pAsp100 compete for binding to pLys250. Interestingly, the strategy in which pLys250 was initially mixed with pAsp100, followed by the addition of fibrinogen, resulted in observable clustering under stoichiometric monomer ratio conditions.

Next, we varied the terpolymer amount to investigate the role of the stabilizing agent in the adhered complex coacervation process (Figure S6). An increase in terpolymer concentration resulted in the formation of smaller coacervate droplets under all the tested preparation protocols. Interestingly, increasing the amount of the stabilization agent led to less clustering, suggesting an obstruction of the complex coacervate–matrix interactions. When we switched to a shorter poly-L-Lysine peptide (pLys100), both complex coacervation and the intermediary matrix formation behavior were significantly hindered despite the high terpolymer amounts, further confirming the importance of the strength of electrostatic interactions between individual components (Figure S7).

**Figure 2:**
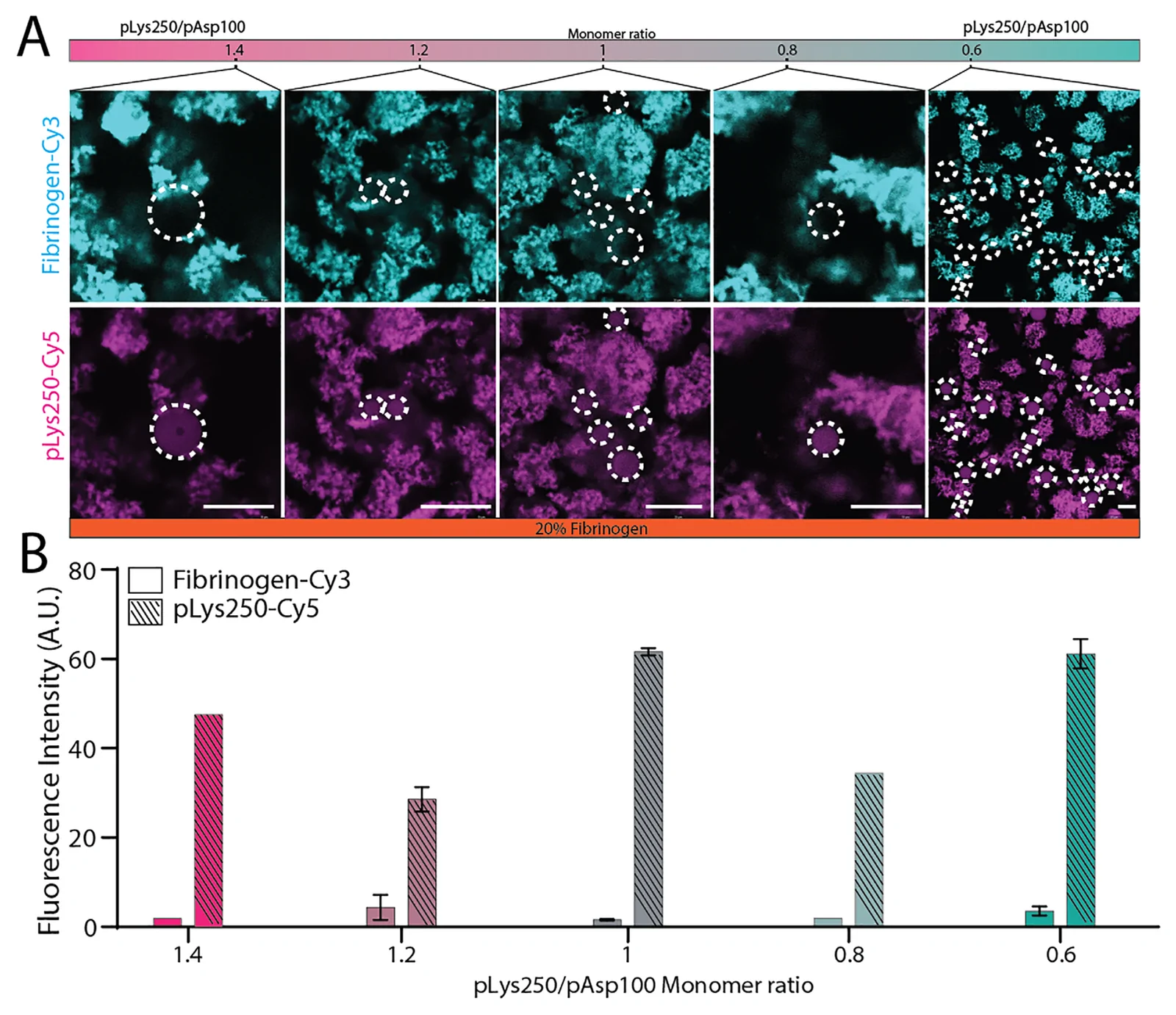
(A) The role of fibrinogen in the adhered complex coacervation process was limited to forming intermediary complexes, regardless of the monomer ratios under the tested conditions. (B) The graphs show the fluorescence signal intensity measurements of fibrinogen-Cy3 and pLys250-Cy5. White dashed circles on the CLSM micrographs indicate the formed complex coacervates between the intermediary complexes (Scale bars: 25 μm) (Fibrinogen was labeled with Cy3, and pLys250 was labeled with Cy5. Cy3 Ex: 550 nm Em: 570 nm, Cy5 Ex: 650 nm Em: 670 nm) (Brightness and contrast were manually adjusted during post-processing for visualization purposes).

Lastly, we investigated whether fibrinogen participates in LLPS beyond forming the intermediary complexes. We followed the order of addition, in which LLPS was triggered by a fibrinogen–pLys250 intermediary complex. The results confirmed that fibrinogen was neither sequestered nor participated in LLPS as a polyelectrolyte across all tested monomer ratios (Figure 2A,B). Furthermore, when different order-of-addition strategies were followed, we observed that fibrinogen can participate in the complex coacervate stabilization process, but again was never sequestered within the coacervates under all tested conditions (Figure S8). Based on the abovementioned observations we therefore followed the pathway, in which the fibrinogen–pLys250 intermediary complex was initially formed, followed by LLPS upon the introduction of pAsp100 at higher total polypeptide and terpolymer concentrations. This resulted in smaller, matrix-adhered, and more densely packed coacervate droplets for further studies.

We also tested various commonly used biomaterials, such as gelatin type b, gelatin type a, or hyaluronic acid in combination with different types of complex coacervate-based artificial cell models, such as quaternary amine-modified amylose and carboxymethyl amylose, or poly(diallyldimethylammonium chloride) (PDDA) and adenosine triphosphate (ATP), but we did not observe stable combinations in which the complex coacervates maintained their structural integrity after the LIFT printing protocol (Figure S9A–C). This can be explained by the absence in these cases of adherence of the coacervates to the biomaterial ink.

### 2.2 Optimization of LIFT Parameters for Adhered Complex-Coacervate Transfer

**Figure 3:**
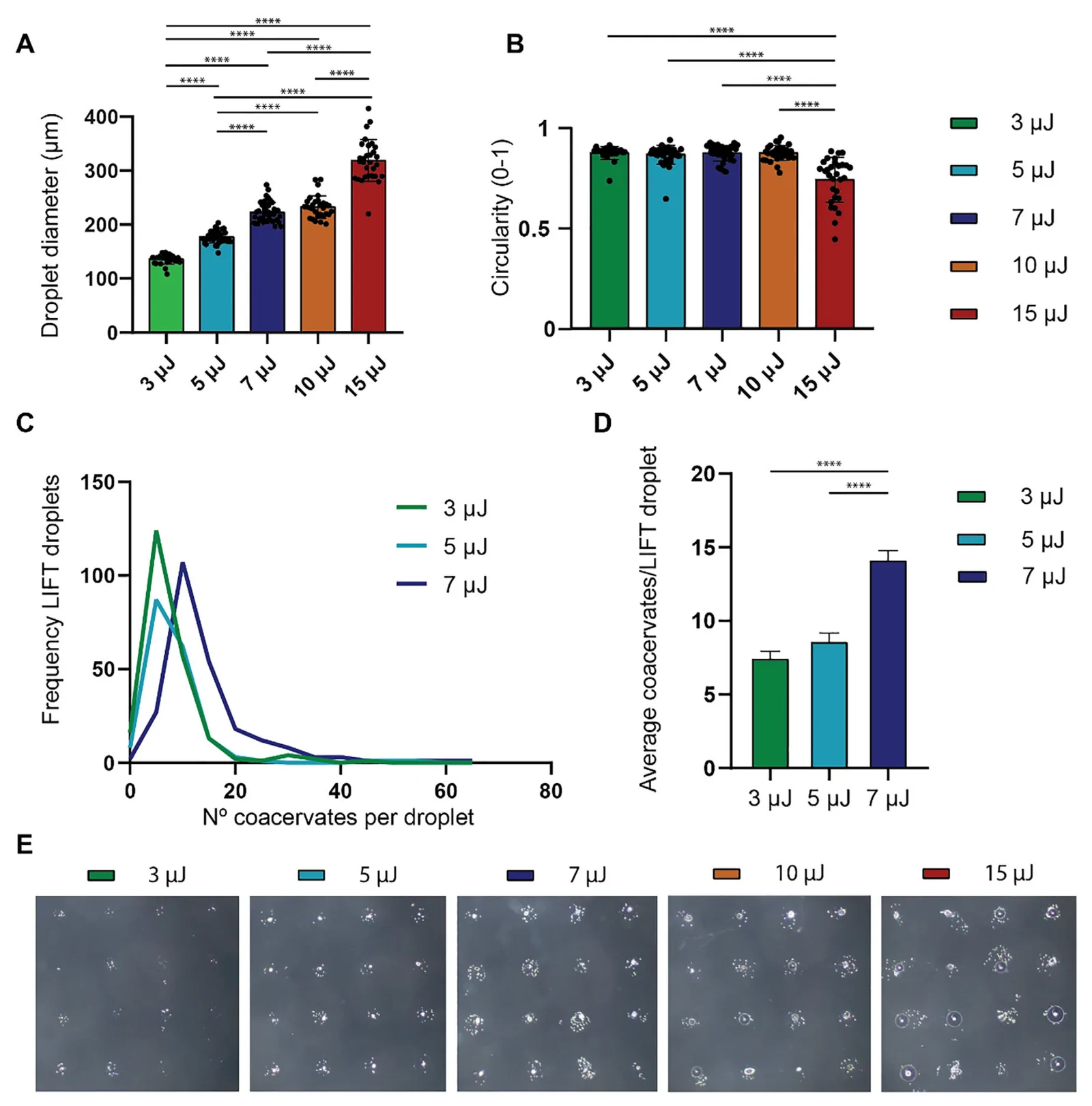
Screening of laser energy used to LIFT-print coacervates in optimal conditions at a constant distance donor receiver (DDR) of 1.5 mm, loading volume of 7 μL, laser energies of 3, 5, 7, 10, and 15 μJ. (A) Droplet diameter at increasing laser energy, (B) circularity of droplets at increasing laser energy, (C) coacervate distribution per droplet at different printing energy levels, (D) average coacervate number per droplet at increasing laser energy, and (E) brightfield microscopy micrographs at increasing laser energy. Statistical analysis one-way Anova with multiple comparisons, significance *** = *p*-value 0.0005 **** = *p*-value 0.0001 (N = 5, n = 20).

After forming the biomaterial ink, we studied its printability with LIFT in a fibrin-thrombin matrix, and post-print stability of the generated structures to determine the optimal printing parameters. Printing the coacervates embedded in fibrinogen onto a fibrin receiver ensures their immobilization through local crosslinking of excess thrombin in the fibrin gel [31]. The starting printing conditions of loading volume, distance donor receiver (DDR), and receiver material composition were based on prior research [19]. We first evaluated the effect of laser energy on the diameter (Figure 3A) and circularity (Figure 3B) of the printed droplets, followed by the effect of laser energy on the coacervate deposition frequency (Figure 3C) and deposition rate (Figure 3D) within LIFT-printed droplets. Furthermore, we compared the morphology of the coacervates between printed and non-printed conditions (Figure S10). The effect of laser energy did not have an effect on coacervate diameter. All LIFT-printed coacervates appeared to be significantly smaller than the non-printed controls, however, the standard deviation of the non-printed controls is larger than the standard deviation found in the printed samples. We hypothesize that due to the inherent process of printing, the larger fraction of coacervates eventually precipitates and that with increased number of pipetting steps, the larger coacervates could shear and break or remain attached to the bottom of the tube. Furthermore, the LIFT printing process could mechanistically bias toward the transfer of the smaller fraction of coacervates due to a lesser disturbance to the homogeneity of the bioink suspension. The diameter of the printed droplets increased consistently with laser energy, from 130 μm at 3 μJ to 300 μm at 15 μJ. We also observed an increase in the standard deviation of both parameters as laser energy increased, due to the material being accelerated at a much faster rate and arriving at the receiver with a higher terminal velocity [24, 32]. Increasing the laser energy at a fixed DDR resulted in increasing the terminal velocity of the jet upon deposition on the receiver substrate. This compromised the integrity of the coacervates in the transferred material as well as that of the receiver surface, resulting in piercing and embedding of the printed droplets in the fibrin matrix (Figure 3E) [24]. The resulting punctures and embedded material beneath the receiver hydrogel surface significantly impaired the analysis of the transferred coacervates. Coacervate quantification is therefore shown only for laser energies of 3, 5, and 7 μJ, where the incidence of receiver punctures remained low or negligible. The distribution of coacervates embedded in each printed droplet increased with laser energy, as expected with increasing droplet diameters. Increasing laser energies have been shown to result in a larger volume of donor material being ejected from the film at constant material viscosities [19]. The distribution of coacervates per droplet followed a Gaussian distribution without achieving normality (Figure S11A). The average coacervate counts at 3 and 5 μJ were very similar, at 13 and 15 coacervates per drop, respectively, whereas the count increased significantly at 7 μJ. Accounting for the number of coacervates transferred per droplet and their distribution across the sample, 7 μJ would seem to be the optimal laser energy. Nevertheless, the droplet diameter increased from 170 to 230 μm between 5 and 7 μJ while substantially increasing the incidence of receiver punctures. Smaller, more consistent droplet transfers that do not damage the receiving surface are advantageous for local delivery and micropatterning of the receiving substrate. Therefore, we chose 5 μJ as the optimal printing laser energy.

**Figure 4:**
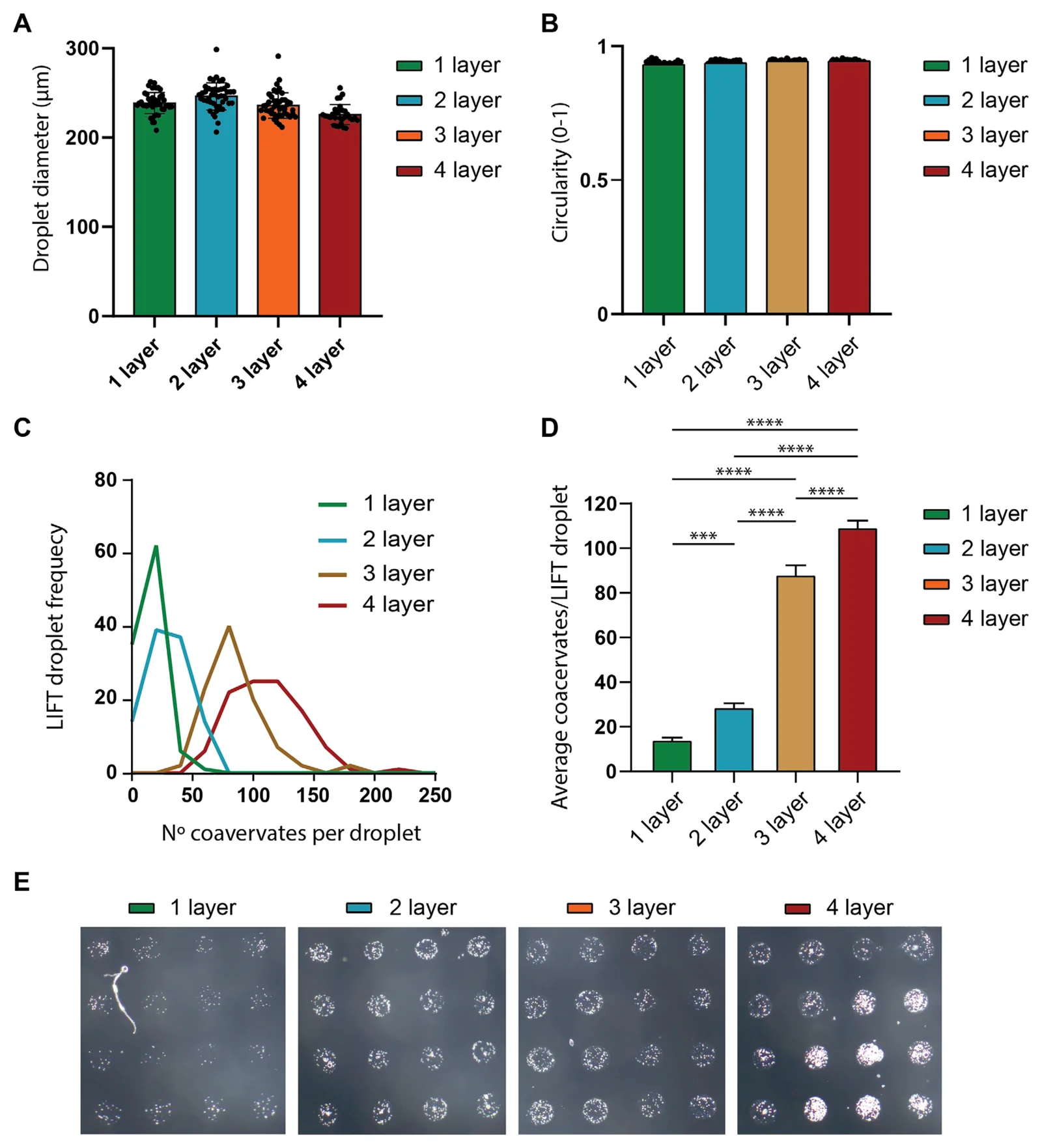
Screening of n° accumulated LIFT printed droplets under optimal conditions at a constant DDR of 1.5 mm, loading volume of 7 μL, laser energy 5 μJ. (A) droplet diameter change at increased accumulated droplet number, (B) circularity of droplets at increasing accumulated droplet number, (C) coacervate distribution per droplet at increasing accumulated droplet number, (D) average coacervate number per droplet at increasing printed layers, and (E) brightfield microscopy micrographs at increasing accumulated droplet number. Statistical analysis one-way ANOVA with multiple comparisons, significance *** = *p*-value 0.0005 **** = *p*-value 0.0001 (N = 5 n = 20).

We subsequently investigated the possibility to accumulate multiple printed droplets containing coacervates at the same spot in order to increase their amount. The accumulation of 2 to 4 LIFT printed droplets did not result in an increase of either diameter (Figure 4A) or circularity (Figure 4B). Upon studying the coacervate distribution per droplet, a gradual shift towards a wider distribution was observed, with 4 accumulated printed droplets being the only sample with a normal distribution (Figure 4C and Figure S11B). We observed that at higher print numbers, the number of coacervates per droplet followed a normal distribution. For the single print sample, the main frequency of coacervates per printed droplet was 15. Upon increasing to two, three, and four accumulated prints, the coacervate average frequency increased to 35, 82, and 110, respectively (Figure 4D). These data was consistent with previously reported distributions of single cells in LIFT printed droplets for biomaterial inks with increasing cell concentrations, and with previous reports on the accumulation of LIFT printed spheroids [22]. Furthermore, increasing accumulated droplets did not result in increased incidence of receiver punctures (Figure 4E) as was observed at laser energies from 7 μJ and upwards (Figure 3E). Therefore, accumulated printed droplets in one location increased the concentration of printed coacervates while maintaining the hydrogel´s surface intact, ensuring consistent delivery of the coacervates.

Based on these findings, it was concluded that the optimal conditions for the LIFT transfer of the coacervates are DDR = 1.5 mm, loading volume of 7 μL, laser energy of 5 μJ, and a print number of 3. The difference in coacervate concentration per droplet increased significantly between 2 and 3 accumulated droplets and between 3 and 4 accumulated droplets, with a more pronounced increase for the former. Furthermore, adding a fourth accumulated droplet required additional resources, bringing the total volume of biomaterial ink containing coacervates to 28 μL for a 4-tiered sample. Therefore, to compromise between printing efficiency and resource use, further experiments were performed by printing three accumulated droplets of coacervates consecutively.

### 2.3 Co-Localization of Different Adhered Complex-Coacervate Populations

**Figure 5:**
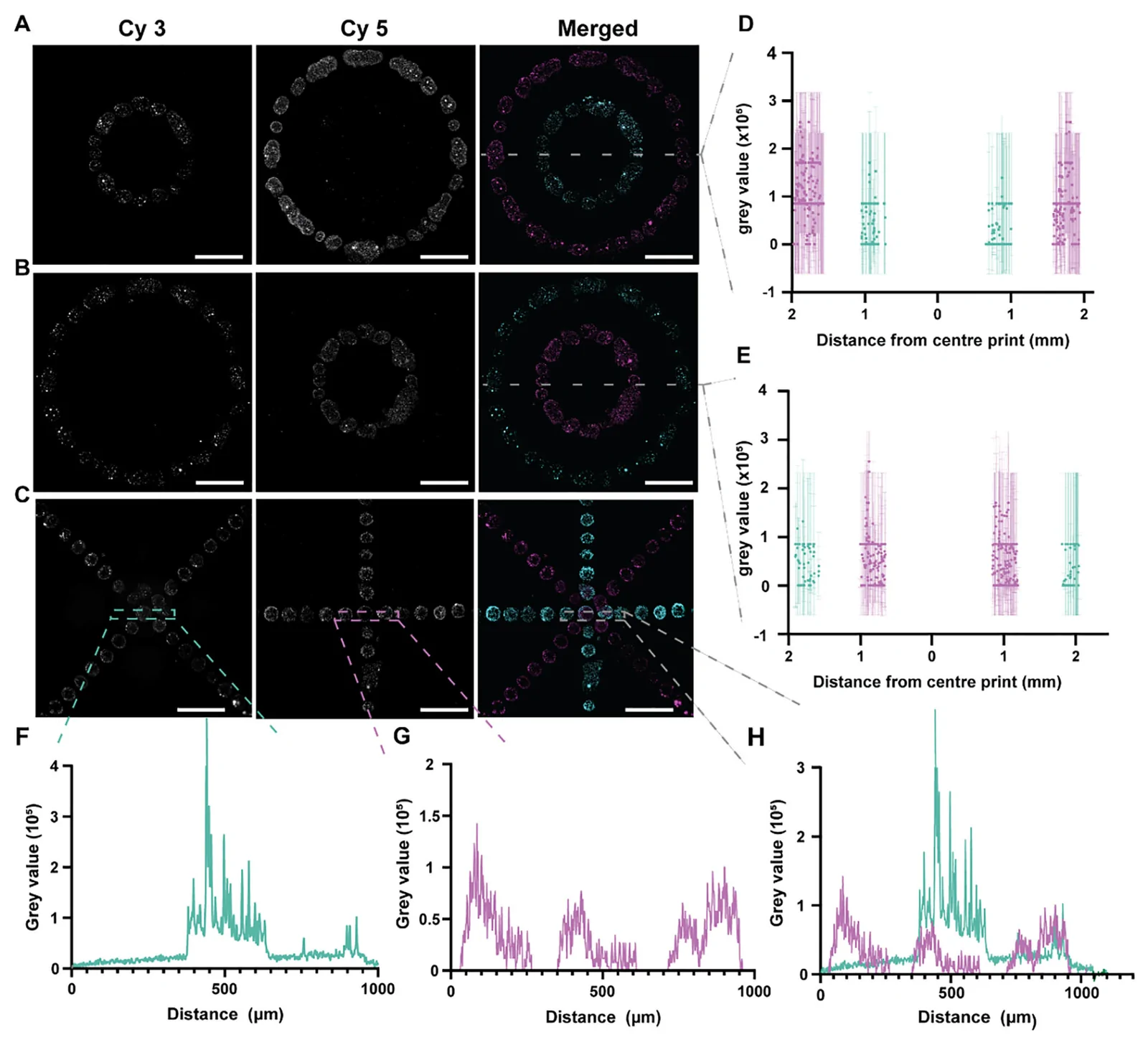
Co-localization of differently labeled coacervate populations via LIFT. Two populations, labeled with either Cy3 or Cy5 dyes, were LIFT printed sequentially. LIFT printing performed at DDR of 1.5 mm, loading volume of 7 μL, laser energy 5 μJ. (A) Cy3-labeled population 2 mm diameter circle, Cy5-labeled population 4 mm diameter circle. (B) Cy5-labeled population 2 mm diameter circle, Cy3-labeled population 4 mm diameter circle. (C) Cy3-labeled population 45° cross Cy5-labeled population upright cross. (D) Coacervate distribution along a centerline on print (A), indicated in the image overlay, shows the location of both population coacervates. (E) Coacervate distribution along a centerline for prints on panel B, as indicated in the image overlay, shows the location of both populations. (F) Distribution of Cy3-labeled coacervates along the centerline. (G) Distribution of Cy5-labeled coacervates along the center line. (H) Distribution of both Cy3 and Cy5-labeled coacervates along the centerline in prints shown on panel C. Prints were imaged with a Thunder microscope and analyzed for coacervate distribution across a centerline using Image J. (Scalebar = 1 mm).

LIFT enables high printing speeds and a high degree of design freedom, which, in turn, makes the technology suitable for multi-material printing [33]. In view of this, two coacervate populations were prepared either labeled with Cy3 or Cy5 dyes and sequentially patterned in two concentric circles Cy3–Cy5 (Figure 5A), Cy5–Cy3 (Figure 5B), and a superposed cross design 45° Cy3 0° Cy5 (Figure 5C). The localization of each coacervate population in the concentric circle designs proved to be mutually exclusive with no overlap between the purple (Cy3) and blue (Cy5) signals (Figure 5D,E). The localization of the coacervates in the droplets was also found to be 2 mm apart for the inner circle and 4 mm apart for the outer circle, according to the design input for those prints. The cross design studied the co-localization of two patterns with a crossing point in its center, where a single droplet overlaps both designs. Signal quantification on the Cy3 channel showed the presence of one LIFT printed droplet (Figure 5F) while the Cy5 quantification showed three consecutive LIFT prints in the same quantified area (Figure 5G). The overlay of the two channels showed precise co-localization of the populations at the interface between the designs (Figure 5H). These results demonstrated the use of LIFT to micropattern diverse and stable populations of coacervates in close proximity, with high design freedom.

### 2.4 Controlled Cargo Uptake and Release on LIFT Printed Adhered Complex-Coacervates

Cargo uptake and release of bioactive components are important features in artificial cell and tissue models. It is therefore important to ensure that these features are retained when integrated into biofabrication strategies.

Controlled cargo uptake can be achieved based on the interactions between the cargo molecules and the receiver carrier, coacervates in this case. Molecular weight and isoelectric point are two main driving forces affecting cargo sequestration efficiency [34, 35]. We therefore tested the uptake of a range of proteins with varying isoelectric point and molecular weight into complex coacervates prepared with different monomer ratios, since the monomer ratio determines the coacervates’ overall charge density. We observed that the isoelectric point was indeed the primary factor determining the sequestration of the tested proteins within coacervate droplets in our system (Figure S12, Table S1). When the isoelectric point of the proteins was above the buffer pH, no cargo sequestration was observed. Yet cargo uptake was not hindered when proteins of different molecular weights were used, provided their respective pI values were below the buffer pH. The highly charged and dynamic environment of the complex coacervates, along with the permeable terpolymer membrane, allowed the sequestration of cargo molecules, including those with a high molecular weight. While these results confirm the effect of electrostatic interactions in controlled cargo sequestration studies, it should be noted that the effective driving force depends on the mechanism used to control cargo sequestration. Studies show that both molecular weight and isoelectric point are important for cargo delivery, where molecular weight creates a physical barrier that the cargo’s surface charge can overcome [36–38].

**Figure 6:**
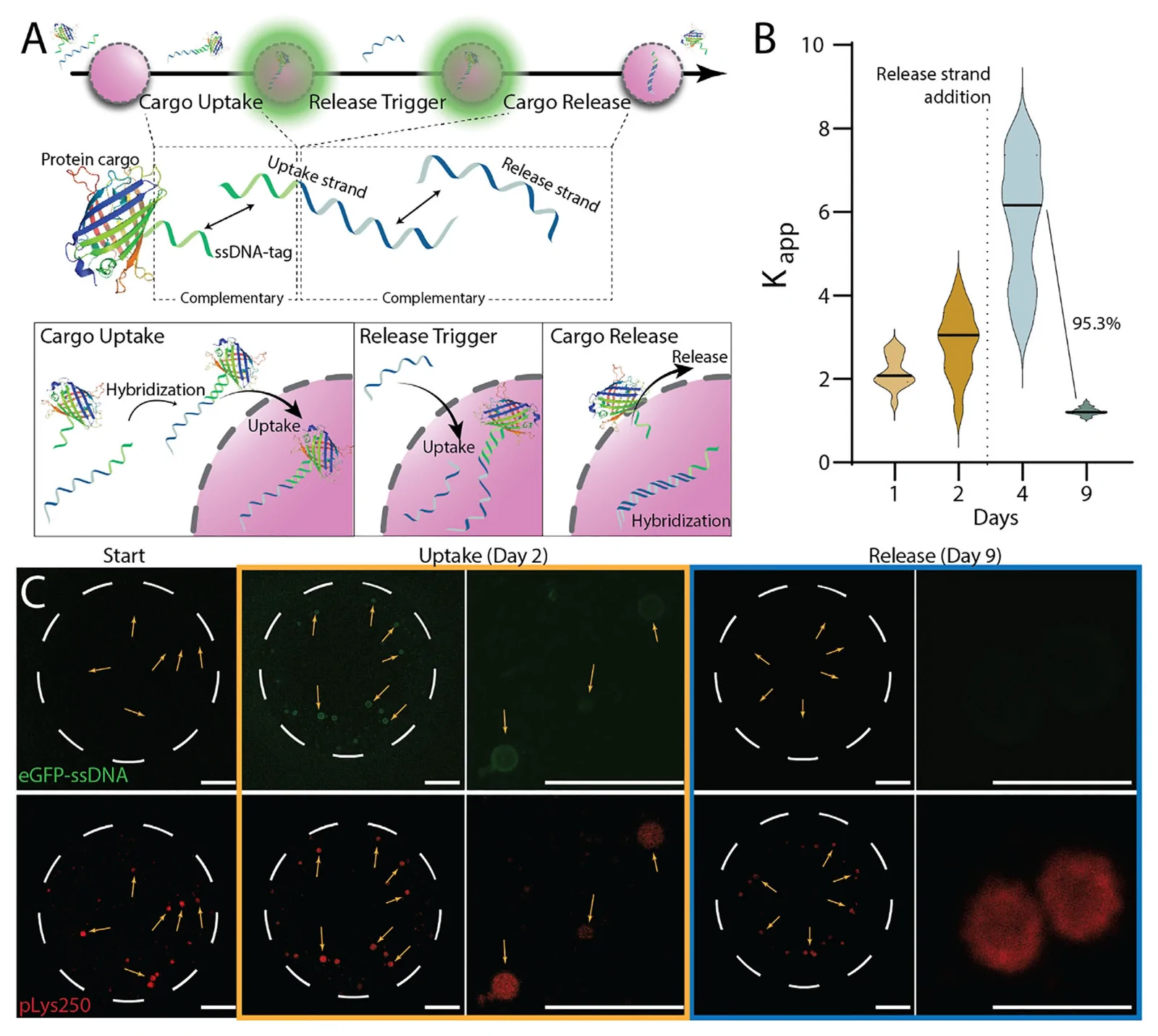
(A) Schematic showing the DNA-mediated uptake and release mechanism. (B) Changes in apparent partition coefficient (K_app_) upon sequential cargo uptake and release. (C) The uptake and release behavior observed by CLSM. Individual coacervate droplets were visualized by the labeled pLys250 component, and the cargo molecule was visualized in the eGFP channel. Dashed circles indicate the LIFT droplet periphery, and orange arrows point to individual coacervates on both channels. Micrographs without dashed circles are the zoomed-in micrographs of the samples. (Green channel: cargo, eGFP, Red channel: coacervate, pLys250-Cy5). (Scale bars: 100 and 25 μm for uptake, 100 and 15 μm for release).

Next, we applied our previously developed DNA-mediated cargo uptake and release strategy [39, 40] to the LIFT printed, adhered complex-coacervate populations. The controlled uptake briefly relies on the interactions between a short single-stranded DNA (ssDNA-tag, 12 bases) modified protein (eGFP) and a longer single-stranded DNA molecule (uptake strand, 32 bases) that is complementary to the 12 bases of the ssDNA-tag. The increase in negative charge upon hybridization enables uptake of the eGFP DNA hybrid. Next, a longer single-stranded DNA molecule (release strand, 32 bases), which is complementary to the uptake strand, is added. This triggers eGFP release by the in situ hybridization of the uptake and release strands (Figure 6A).

We monitored the uptake and release behavior of the fluorescent cargo molecule (eGFP-ssDNA-tag) by measuring changes in fluorescence intensity in LIFT printed adhered complex-coacervate populations, following the above-described protocol, and calculated the apparent partition coefficient (K_app_) (Figure 6B). The increase in the K_app_ values between days 1 and 2 indicated that the uptake behavior takes multiple days. This result is significantly slower than the reported studies using the same method in a liquid matrix and likely reflects slower diffusion dynamics in viscous environments [39]. Interestingly, the K_app_ value was further increased, reaching 5.826, on day 4, despite the addition of the release strand a day before. This result further suggested that a similar slow diffusion behavior applies to the release strand as well, where strand displacement had not yet become the dominant process. On day 9, however, strand displacement had already occurred, and the cargo release behavior was significantly prominent, with K_app_ decreased to 1.226, approaching unity. Consistent with this change in partitioning, the loss of excess partitioning can be calculated as 95.3%, considering K_app_ = 1 representing no partitioning. This process was furthermore confirmed by confocal laser scanning microscopy (Figure 6C).

These findings place the presented in situ formed, LIFT printed, and functional coacervate system within the broader development of dynamically regulated complex coacervates. Recent polymerization-induced electrostatic self-assembly (PIESA) methods, for example, couple the in situ formation of charged polymers to coacervation [41, 42]. Similarly, enzymatically regulated systems generally benefit from reaction-driven changes in the environment (e.g., pH, net charge) to control coacervate formation and subsequent behavior [43, 44]. These approaches primarily regulate coacervate behavior, while functionality is driven by changes in mixture composition. In contrast, in our system, the coacervate architecture is preserved while the cargo composition is remodeled through DNA strand displacement. The multi-day sequestration behavior and subsequent release functionality indicate that programmable molecular exchange can be sustained in a viscous, matrix-associated coacervate environment. In this manner, the LIFT printed system combines spatial control over coacervate distribution with temporal control over the cargo dynamics, demonstrating that programmable transport functions can be maintained in patterned, matrix-adhered coacervate-based artificial cell populations.

## 3 Conclusions

We have successfully demonstrated LIFT printing of complex coacervate-based artificial cells using an adhered biomaterial ink preparation method. The adhered complex-coacervation strategy allowed the coacervate droplets to be integrated into a fibrinogen matrix, which then crosslinked into fibrin upon LIFT printing onto a fibrin-thrombin surface. This allowed us to maintain precise localization of coacervate populations within LIFT printed droplets by preventing sinking or floating in a low-viscosity matrix. The in situ crosslinking allowed maintaining hydrated conditions for the coacervate droplets on the receiver surface, which was crucial for preserving their structural integrity against rapid drying. Furthermore, we successfully patterned different populations of coacervate droplets using a combination of LIFT printing parameters that avoided injecting the coacervates into the hydrogel layer while maintaining their structural integrity post-printing. Accumulating multiple LIFT printed droplets significantly increased the number of deposited coacervates per LIFT printed droplet in a controlled manner, without further compromising either receiver or coacervate integrity. Differently labeled populations of coacervates were co-patterned in complex geometries, with mutually exclusive or minimally overlapping distributions, allowing the study of multi-material printing capabilities. Lastly, coacervates remained functional after LIFT printing; cargo-loaded coacervates preserved their cargoes during printing, and a successful model cargo uptake and release process demonstrated that coacervates remain intact and functional post printing.

LIFT is therefore a suitable enabling technology for reproducible, reliable micropatterning of coacervate-based artificial cells in complex geometries while preserving biologically relevant properties and functionality.

## 4 Materials and Methods

### 4.1 Materials

Poly-L-lysine hydrobromide (pLys250, mw: 52 kDa) and poly-(L-aspartic acid) sodium salt (pAsp100, mw: 14 kDa) were purchased from Alamanda Polymers. Fibrinogen (bovine plasma), glucose oxidase (GOx), horseradish peroxidase (HRP), and bovine serum albumin were purchased from Sigma–Aldrich. Terpolymer, mPEG-p(CL-g-TMC)-pGlu was prepared and purified as previously reported [45]. Cyanine5 (Cy5)-labeled pLys250 was prepared by mixing sulfo-cyanine5-NHS ester (Lumiprobe) and pLys250 in a 5:1 molar ratio. The reaction was carried out in 1 m sodium bicarbonate buffer at pH 8.3 for 1 h under constant shaking. After quenching the reaction by adding 4% (v/v) 1 m Tris, the conjugate was dialyzed extensively against PBS for 2 days, refreshing the solution twice daily. DNA-DBCO was purchased from IDT integrated DNA technologies. The Ti/Au absorbing layer-coated donor substrates were purchased from Poietis Biosystems (France). AG86-sBit99, AG29-dogcatch LBit, sBitP7, and LBitP3 were kindly provided by Alexander Stephany and Fabienne Buijs.

### 4.2 Coacervate and Biomaterial Ink Preparation

Terpolymer-stabilized polypeptide-based complex coacervates were prepared by mixing pLys250 (10 mm, PBS) and pAsp100 (10 mm, PBS) in various ratios. The ratio of monomeric concentrations between pLys250 and pAsp100 is called the monomer ratio, indicating the ratio of total monomer amounts in the final mixture. Five different monomer ratios, net positive (1.4) with highest monomeric concentration of pLys250, mildly positive (1.2) with higher pLys250 monomeric concentration than pAsp100, neutral (1) with equal monomer concentrations of pLys250 and pAsp100, mildly negative (0.8) with higher pAsp100 monomeric concentration than pLys250, and net negative (0.6) with highest monomeric concentration of spAsp100, were prepared by mixing polypeptides in an Eppendorf tube in a thermomixer (Eppendorf) at 1500 rpm, for 6 min at room temperature. The terpolymer stabilization was carried out by adding 2% (v/v) terpolymer (25 mg/mL in DMSO) after 6 min, followed by 30 sec of shaking. For washing, the prepared samples were centrifuged at 200 × g for 2 min. The supernatant and the precipitate were separated, and the precipitate was resuspended in PBS by gentle pipetting.

For biomaterial ink optimization, coacervates with different monomer ratios were prepared as described, with fibrinogen (10 mg/mL in PBS) added at a final concentration of 20% (v/v). The order of fibrinogen addition was varied depending on the preparation protocol. When forming intermediary complexes, either pLys250 or pAsp100 was first premixed with fibrinogen, followed by the addition of the remaining polypeptide. When the formation of the intermediary complex was restricted, fibrinogen was added to the polypeptide mixture after 1 min of shaking. The effect of the order of addition was evaluated by considering both the size and the number of the coacervates.

### 4.3 LIFT Printing

LIFT printing was performed accordingly to the previously published protocol [19]. In brief, an NGB-R 4D bioprinter (Poietis Biosystems, Bourdeaux, France) was used. Printing was performed using a donor slide film composed of 2 μL 0.1% Bovine Serum Albumin and 7 μL biomaterial ink. LIFT printing was performed at a fixed Distance Donor Receiver of 1.5 mm and a laser power of 3, 5, 7, 9, or 15 μJ, indicated in the figure legends. Printing was performed in 12-well plate standard cell culture plasticware, to center the prints, inserts of 8 mm diameter, 800 μm height were printed with a FormLabs 3b+ Stereolithography printer using BioMed Clear resin (FormLabs, USA) following the manufacturer´s protocol for both printing and post-curing. The inserts were then autoclaved prior to loading with 60 μL 20 mg/mL Fibrin hydrogel for LIFT printing. Fibrin hydrogels were prepared with 20 mg/mL Fibrinogen (human-plasma, ThermoFischer) and Thrombin (ThermoFischer) at a final concentration of 1 U/mL.

LIFT printed droplets and the coacervates present within the LIFT printed droplets were imaged with an Olympus BX53 for brightfield images and a Thunder microscope for fluorescent images.

### 4.4 DNA-Mediated Cargo Uptake and Release

#### 4.4.1 His-eGFP-N_3_-ssDNA Conjugation

The production of hexahistidine tagged azido-functional eGFP (His-eGFP_Y151pAzF) is described in the Supporting Information. The conjugation was carried out by mixing 1.5 equimolar excess of DNA-DBCO with His-eGFP-N_3_ in a thermomixer (Thermo Fisher Scientific). The reaction mixture was incubated overnight at 4°C at 500 rpm in an amber-colored DNA LoBind Eppendorf tube. Excess DNA-DBCO was removed by anion-exchange chromatography using a proFIRE system (Dynamic Biosensors). Over 160 μL of the reaction mixture was loaded onto a 1 mL (26 × 7.0 mm) column packed with a hydrophilic porous polymer bead matrix. Elution was performed using a linear NaCl gradient at a flow rate of 1 mL min^−1^, starting with low-salt buffer A (50 mm sodium phosphate, 150 mm NaCl, pH 7.2) and increasing the contribution of high-salt buffer B (50 mm sodium phosphate, 1 M NaCl, pH 7.2) over 16 min to a final 70:30 ratio of buffer A to buffer B. Presence of DNA or protein-DNA conjugates was measured by a detector at 260 nm. This procedure resulted in the separation of non-conjugated eGFP, the eGFP–DNA conjugate, and free DNA–DBCO (Figure S13). Fractions containing the eGFP–DNA conjugate were pooled, concentrated, and buffer-exchanged using Amicon Ultra centrifugal filters (10 kDa MWCO; Millipore). Samples were centrifuged at 12 000 × g for 10 min per load, washed 3 times with 1X PBS, and recovered by reverse spin at 2000 × g for 2 min. Protein concentration was determined by measuring absorbance at 280 nm using a NanoDrop spectrophotometer and a molar extinction coefficient of 25 900 M^−1^ cm^−1^.

Conjugation was confirmed by native polyacrylamide gel electrophoresis (Native-PAGE; Figure S14). For each condition, 2 μg of protein in 1X PBS was mixed 1:1 (v/v) with non-reducing 2X Native-PAGE sample buffer (62.5 mm Tris-HCl, 20% glycerol, 0.01% bromophenol blue, pH 6.8). Samples were loaded onto a pre-cast 4%–20% Mini-PROTEAN TGX gel (Bio-Rad) and run in pre-cooled 1X Tris/Glycine buffer (25 mm Tris, 192 mm glycine, pH 8.3) at 90 V for 90 min in an ice bath. Gels were imaged sequentially without staining (eGFP fluorescence), after staining with SYBRTM Gold nucleic acid gel stain (DNA; Invitrogen), and after staining with Bio-Safe Coomassie G-250 protein stain (Bio-Rad) using an Amersham ImageQuant 800 system (Cytiva). Image analysis was performed using ImageJ v1.53t.

The ssDNA sequences used for controlled uptake and release studies are shown in Table 1.

The uptake and release process was based on a slightly modified previously published version. Briefly, 7 μm protein-ssDNA conjugate was incubated with 10 μm UPT strand in an ice bath for 1 h prior to the experiments. This complex was added to the LIFT printed coacervate populations as cargo at final concentrations of 205 nm protein-ssDNA conjugate and 307 nm UPT strand. Following this, the REL strand was added to the system at a final concentration of 533 nm. All samples were incubated at 4°C until imaging. Samples were imaged with a Leica SP8 confocal laser scanning microscope (CLSM). Ex: 488 nm, Em: 507 nm. All the buffer conditions were switched to PBS supplemented with 5 mm MgCl_2_ for the DNA-mediated uptake/release studies. The measurements were conducted on at least three independent LIFT printed droplets containing coacervate populations, printed as separate samples, and at least 5 coacervate droplets were selected from each population for measurement.

K_app_ was calculated as the cargo fluorescence signal intensity inside the coacervate relative to the surrounding media.

### 4.5 Statistical Analysis

One-way ANOVA, along with multiple comparisons, was conducted using GraphPad Prism version 10 software. Results were considered significant when *p* < 0.01. Statistical significance is indicated as *** = *p*-value 0.0005, **** = *p*-value 0.0001. The corresponding figure legends indicate the sample size (n).

## Supplementary Material

Additional supporting information can be found online in the Supporting Information section.

SUPPLEMENTARY INFORMATION

## Figures and Tables

**Table 1:** ssDNA handles for the controlled uptake and release studies.

Name	Sequence
DNA-DBCO	5’-/5DBCON/ GACGGAACTGAC-3’
UPT	5’-GCATACGACTCAC ACACCACCACCGCAACCAC-3’
REL	5’-GTGGTTGCGGTGG TGGTGTGTGAGTCGTATGC-3’

## Data Availability

The data that support the findings of this study are available from the corresponding author upon reasonable request.
