# Supplementary material for: Patterning of Artificial Cell Populations Via Laser Induced Forward Transfer for Controlled and Localized Cargo Uptake and Release
Source: Small. Author manuscript; Available in PMC 2026 Nov 4. (PMC7619699; doi:10.1002/smll.76083)
Supplement: Supplementary file 1 [file EMS221781-supplement-SUPPLEMENTARY_INFORMATION.pdf]

SUPPLEMENTARY INFORMATION

Patterning of Artificial Cell Populations via Laser Induced Forward Transfer for Controlled and Localized Cargo Uptake and Release

Yiğitcan Sümbelli[1 γ], Núria Ginés Rodriguez[2,3 γ], Lars J.M.M. Paffen[1], Arjan Hazegh Nikroo[1], Riccardo Levato [2,3,4], Jan C.M. van Hest[1*], Jos Malda[2,3,4*]

[1]*Department of Biomedical Engineering, Institute for Complex Molecular Systems, Eindhoven University of Technology, Eindhoven, The Netherlands*

[2]*Department of Orthopedics, University Medical Center Utrecht, Utrecht University, Utrecht, The Netherlands.*

[3]*Regenerative Medicine Center Utrecht, Utrecht, The Netherlands.*

[4]*Department of Clinical Sciences, Faculty of Veterinary Medicine, Utrecht University, Utrecht, The Netherlands*

* Corresponding authors: J.C.M.v.Hest@tue.nl and j.malda@umcutrecht.nl

[γ] Contributed equally

ORCID is and Emails:

Yiğitcan Sümbelli: https://orcid.org/0000-0002-5917-9581 y.sumbelli@tue.nl

Núria Ginés Rodriguez: https://orcid.org/0000-0001-9218-5759 n.ginesrodriguez-2@umcutrecht.nl

Lars J.M.M. Paffen: https://orcid.org/0009-0002-0218-6403 l.j.m.m.paffen@tue.nl

Arjan Hazegh Nikroo: https://orcid.org/0009-0003-1774-3409 a.hazegh.nikroo@tue.nl

Riccardo Levato: http://orcid.org/0000-0002-3795-3804 r.levato-2@umcutrecht.nl

Jan C.M. van Hest: https://orcid.org/0000-0001-7973-2404 j.c.m.v.hest@tue.nl

Jos Malda: https://orcid.org/0000-0002-9241-7676 j.malda@umcutrecht.nl

Molecular biology and cloning

Restriction enzymes, polymerases, ligases, and related reaction buffers were acquired from New England Biolabs (NEB). DNA sequences were ordered via Integrated DNA Technologies (IDT). The eGFP protein construct was codon optimized for *E. coli* using IDT's codon optimization tool. The gBlock dsDNA fragment and vector pET28a were double-digested using restriction enzymes NcoI-HF and XhoI. Following ligation into the linearized vector backbone using T4 ligase, the resulting construct was verified using Sanger sequencing (Azenta GENEWIZ) and transformed into BL21(DE3) *E. coli* cells (Novagen).

The plasmid pEVOL-pAzF, encoding for the orthogonal aminoacyl-tRNA synthetase–tRNA pair to incorporate p-azido-L-phenylalanine (pAzF) via amber stop codon suppression, was a kind gift from Peter Schultz (Addgene plasmid # 31186).

Expression and purification of eGFP_Y151pAzF

His-eGFP_Y151X-Strep and a tRNA synthetase/tRNA pair for the *in vivo* incorporation of pAzF, encoded in pET28a and pEVOL plasmids, respectively, were co-transformed in *E. coli* BL21(DE3) (Novagen). An overnight culture was inoculated in 1 L of 2YT medium supplemented with kanamycin (50 µg mL-1), chloramphenicol (25 µg mL-1) and pAzF (2 mM). The resulting large culture was grown at 37 °C and 150 rpm to an OD600 of 0.4, after which the expression of the tRNA synthetase/tRNA pair was induced with L-arabinose (0.02% w/v). An hour later, the expression of His-eGFP_Y151X-Strep was induced using IPTG (0.5 mM). Following overnight expression at 18 °C at 150 rpm, the cells were harvested by centrifugation at 4 °C and 10.000 × g for 10 min. The cell pellets were resuspended in 10 mL/g cell pellet buffer A (50 mM Tris, 300 mM NaCl, 30 mM imidazole, 5% glycerol, pH 8.0), supplemented with 1 µL/g cell pellet benzonase nuclease (Novagen) and lysed by three runs through an EmulsiFlexC3 high pressure homogenizer (Avestin) at 15.000 psi. Cell debris was removed by centrifugation at 4°C and 40.000 × g for 20 min, after which the His-tagged protein was purified using Ni-NTA affinity chromatography (His-Bind Resin, Novagen). The lysate was loaded onto the His-Bind resin and washed with 2 column volumes (CV) of buffer A to remove nonspecifically bound products. Subsequently, the His-tagged protein was eluted from the column using buffer B (50 mM Tris, 300 mM NaCl, 250 mM imidazole, 5% glycerol, pH 8.0). The collected elution fraction was then loaded on a Strep-Tactin XT high-capacity column (Iba Lifesciences). After 2 CV washes using Buffer W (100 mM Tris, 150 mM NaCl, 1 mM EDTA, pH 8.0), the protein was eluted in separate column volumes using freshly prepared buffer BXT (100 mM Tris, 150 mM NaCl, 1 mM EDTA, 50 mM biotin, pH 8.0). The elution fraction was analyzed using SDS-PAGE (4–20% Mini-PROTEAN TGX Precast Protein Gel, Bio-Rad) and concentrated using Amicon Ultra Centrifugal Filters (3 kDa MWCO, Millipore) (Figure S13). Protein concentrations were determined by absorbance measurements at 280 nm using an ND-1000 spectrophotometer (Thermo Scientific) and extinction coefficients obtained from the Expasy ProtParam online tool (Table S2). Protein aliquots were flash-frozen in liquid nitrogen and stored at -80 °C.

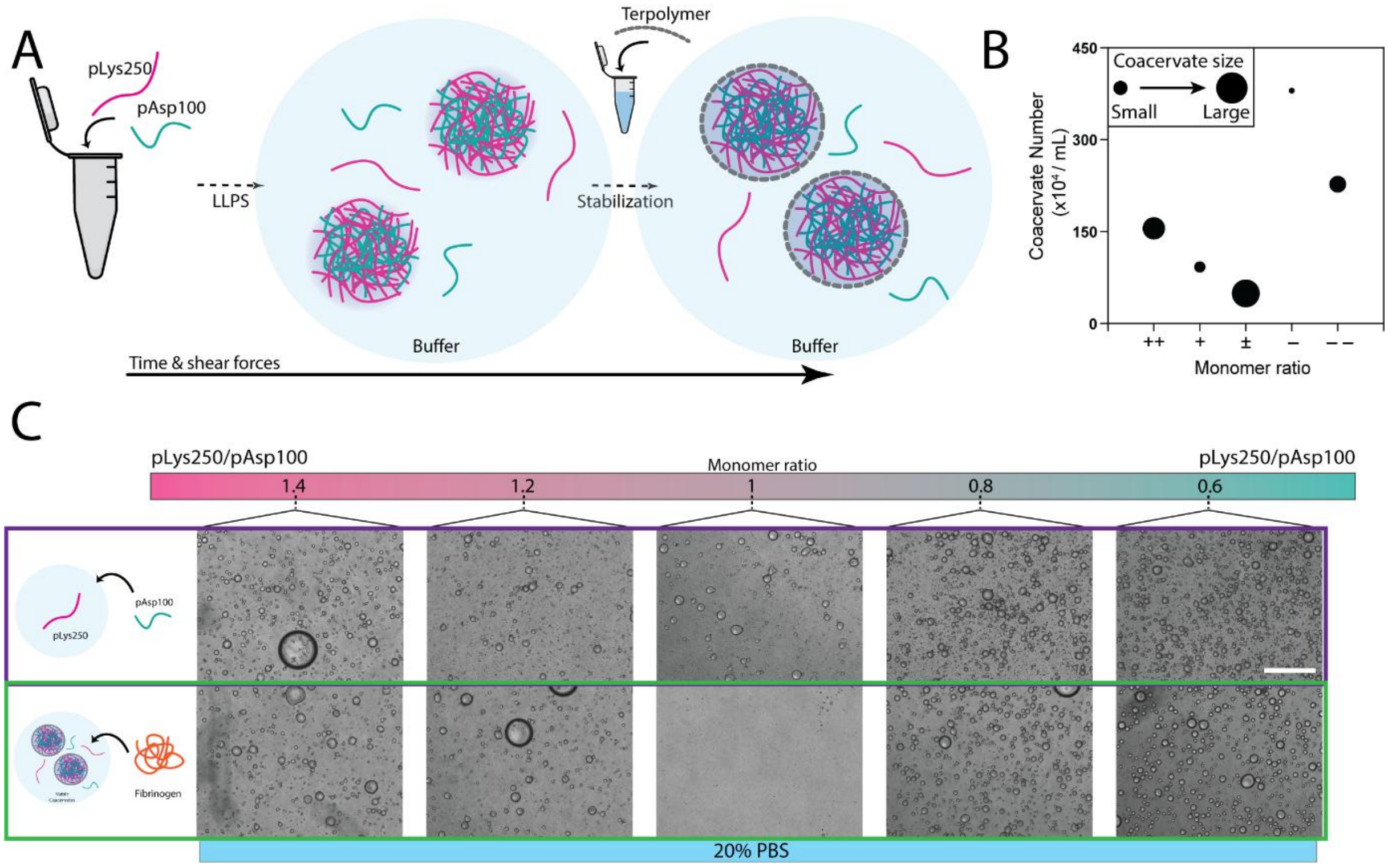

Figure S1. *Terpolymer-stabilized complex coacervation between pLys250 and pAsp100 at different monomeric ratios.* ***(A)*** *The schematic shows the LLPS and the subsequent stabilization process.* ***(B)*** *The size and number of formed coacervate droplets depended on the monomer ratio, and* ***(C)*** *mixing stabilized coacervate droplets with a fibrinogen matrix, following a traditional bioink preparation approach, did not significantly change the number or size of the coacervates. (Scale bar: 50 µm)*

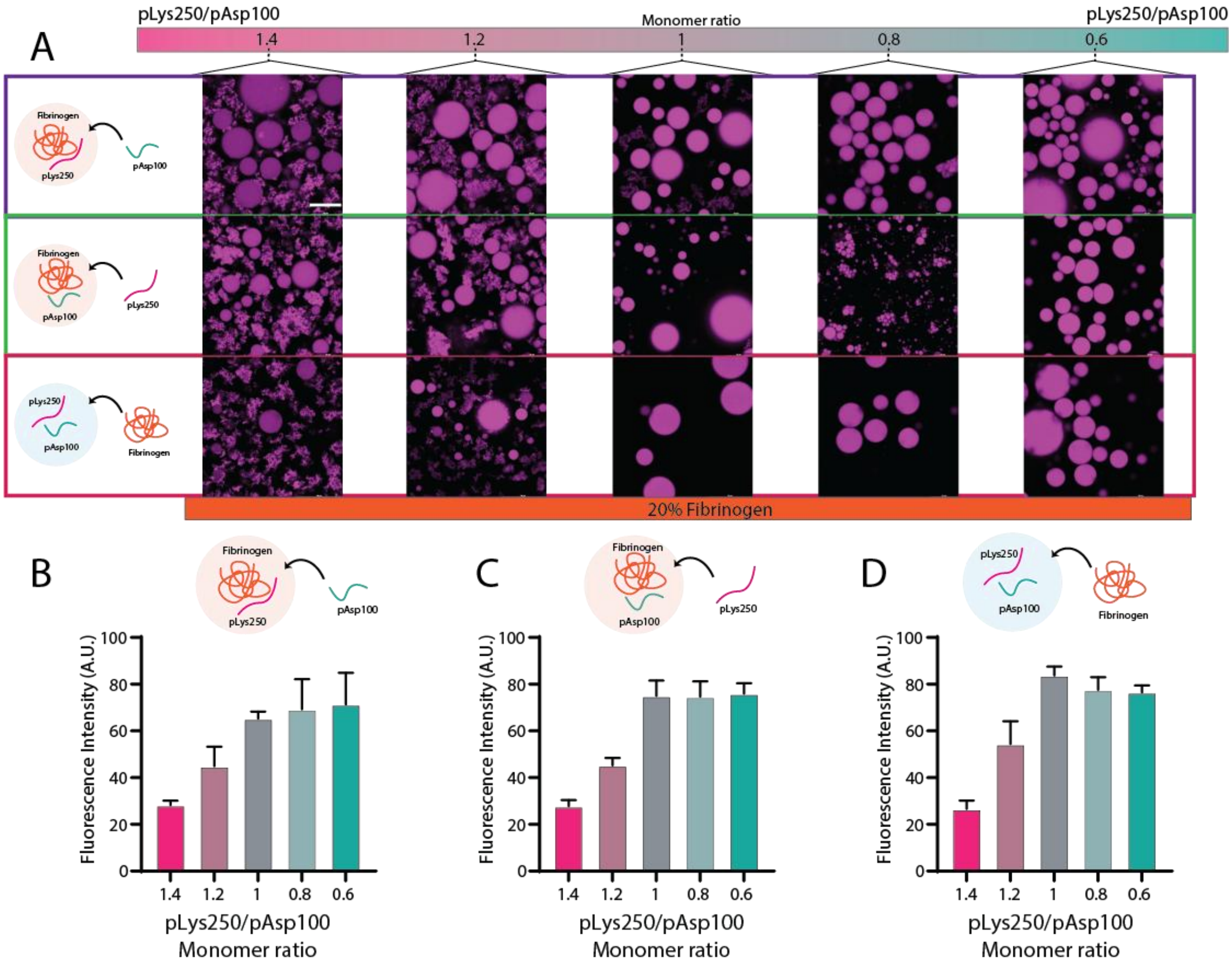

Figure S2. ***(A)*** *CLSM micrographs of pLys250-Cy5 containing coacervates, prepared at different monomer ratios, by following three preparation pathways. The fluorescent signal intensities were digitally increased post-imaging, for visualization purposes.* ***(B-D)*** *Graphs show the pLys250-Cy5 signal intensity measurements within coacervate droplets. Graphs show mean ± SEM. (Scale bar: 25 µm)*

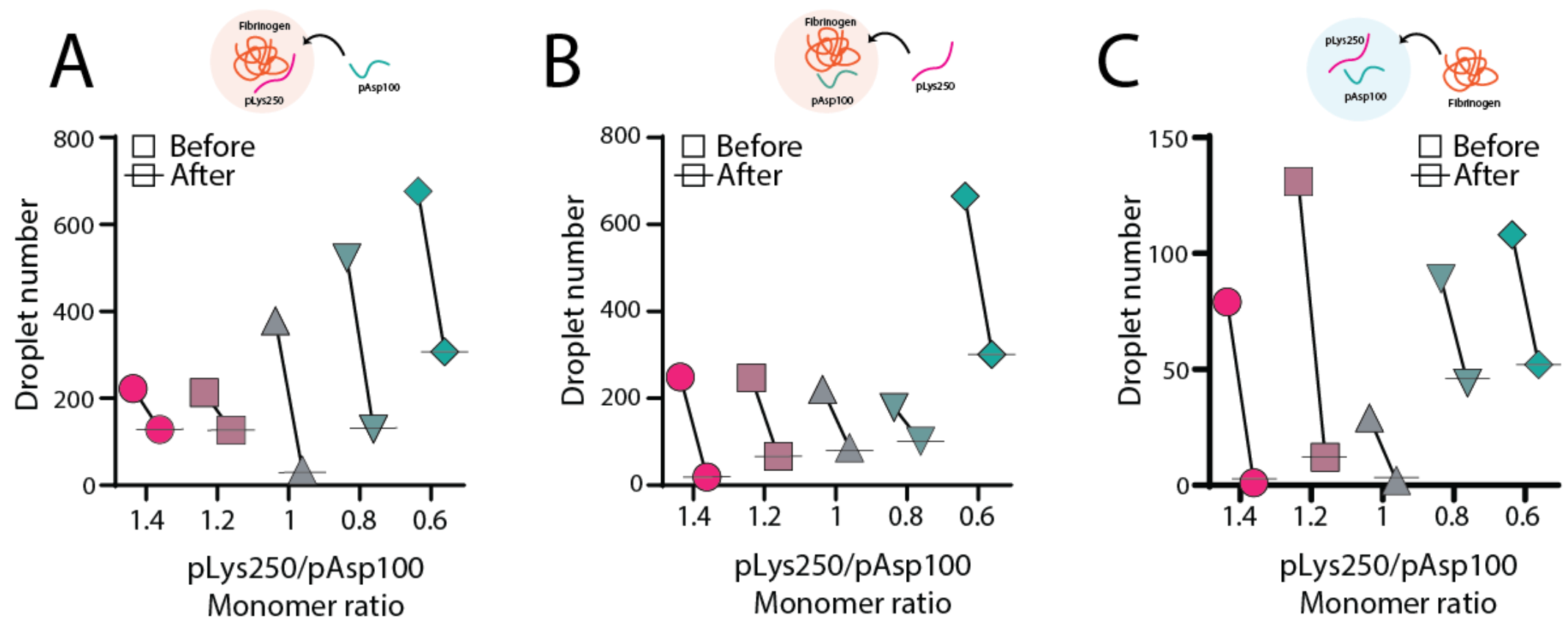

Figure S3. *Graphs showing the changes in coacervate droplet number before and after centrifugation and resuspension steps.* ***(A)*** *When fibrinogen was premixed with pLys250,* ***(B)*** *when fibrinogen was premixed with pAsp100, and* ***(C)*** *when pLys250 and pAsp100 were premixed prior to fibrinogen addition.*

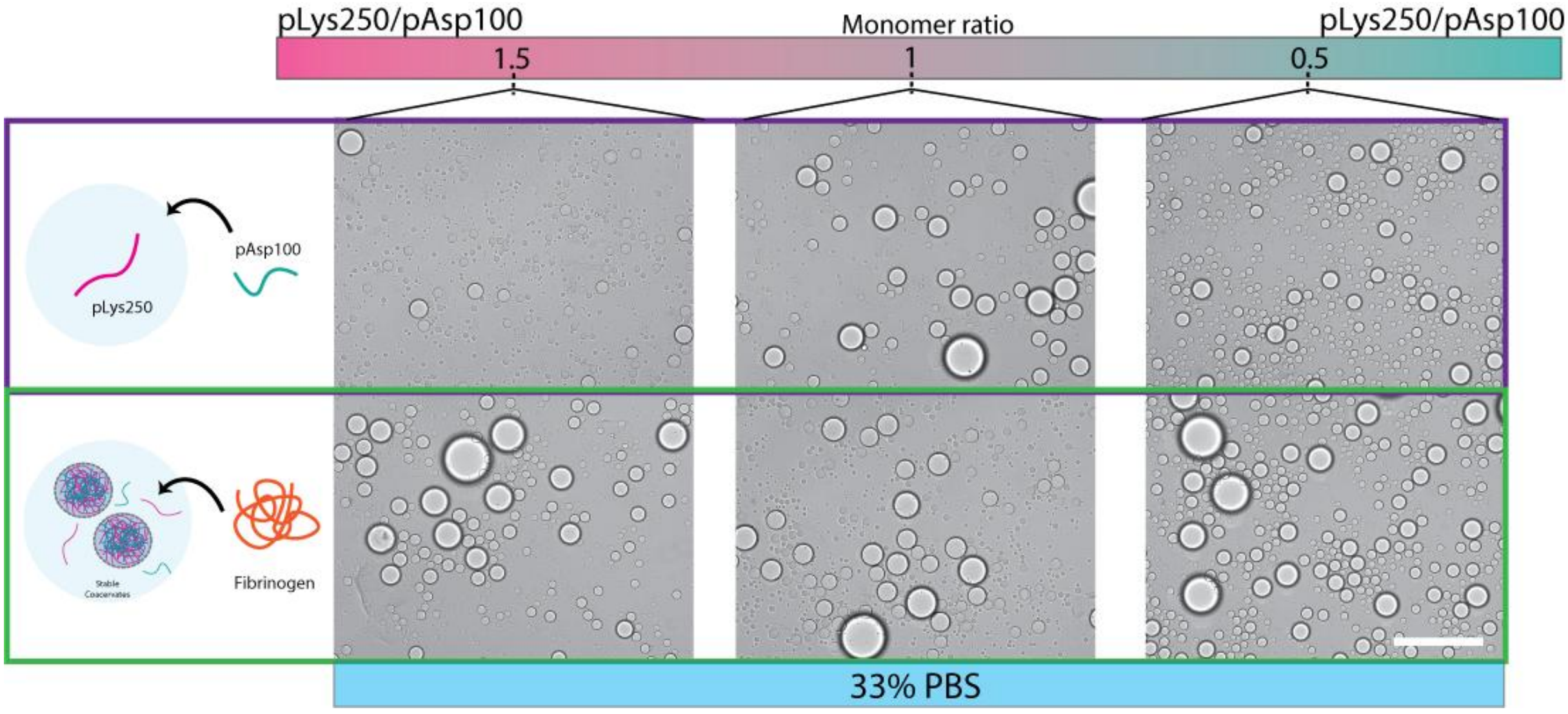

Figure S4. *Terpolymer-stabilized complex coacervation between pLys250 and pAsp100 at 5 mM polypeptide concentrations. (Scale bar: 50 µm)*

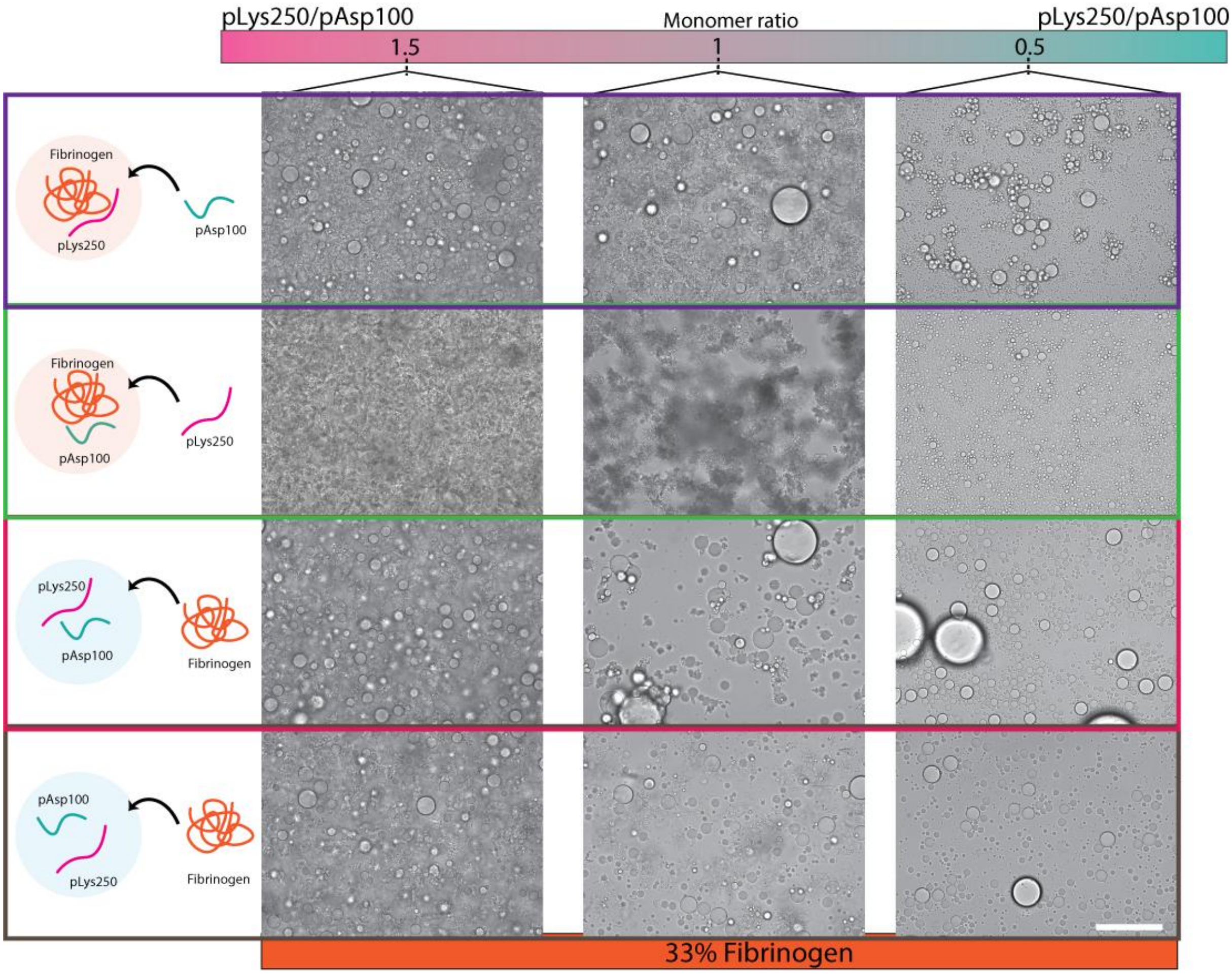

Figure S5. *Changes in the order of component addition led to variations in coacervate number, size, and stability at 5 mM polypeptide concentrations. Each horizontal block consists of different monomeric ratios of a preparation protocol. The purple block (top row) shows the formation of the intermediary complex between fibrinogen and pLys250, followed by the addition of pAsp100. The green block shows the fibrinogen-pAsp100 mixture combined with pLys250 to trigger LLPS. Red and black rows show the addition of fibrinogen during the LLPS process between pLys250 and pAsp100. (Scale bar: 50 µm)*

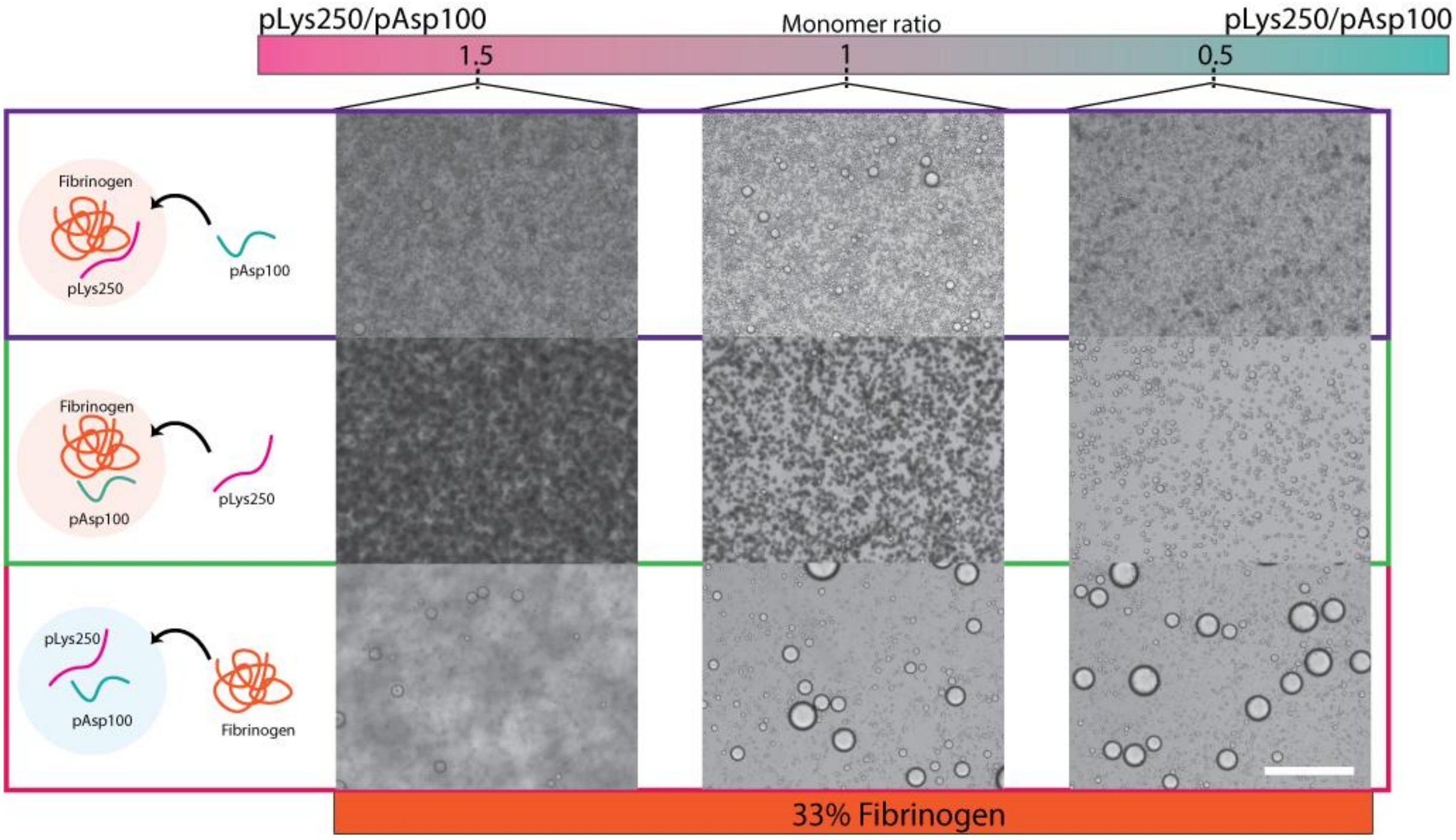

Figure S6. *Changes in the order of component addition led to variations in coacervate number, size, and stability at 5 mM polypeptide concentrations for 4% (v/v) terpolymer concentrations, for the combination of pLys250 and pAsp100. (Scale bar: 50 µm)*

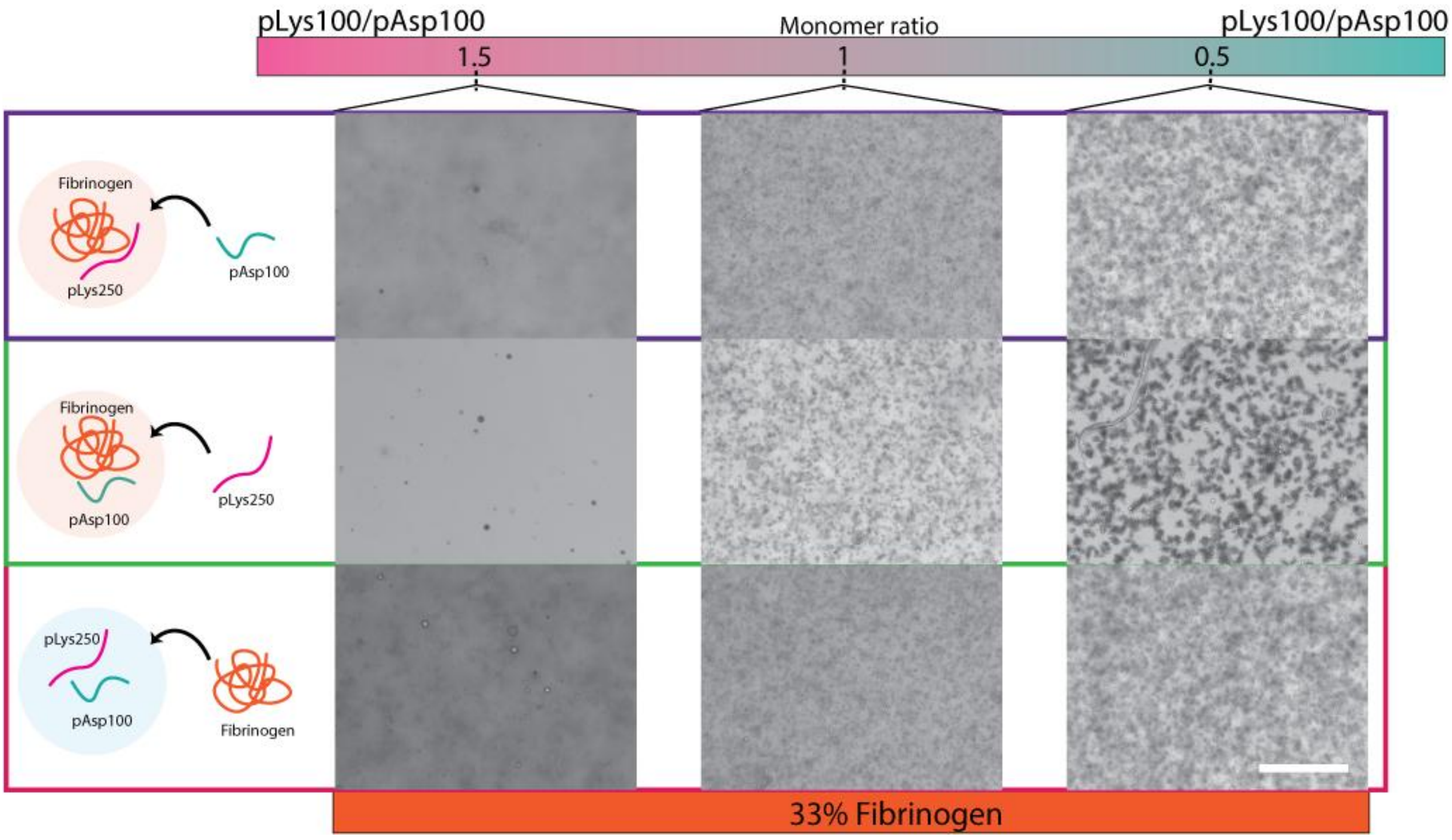

Figure S7. *Changes in the order of component addition led to variations in coacervate number, size, and stability at 5 mM polypeptide concentrations for 4% (v/v) terpolymer concentrations, for the combination of pLys100 and pAsp100. (Scale bar: 50 µm)*

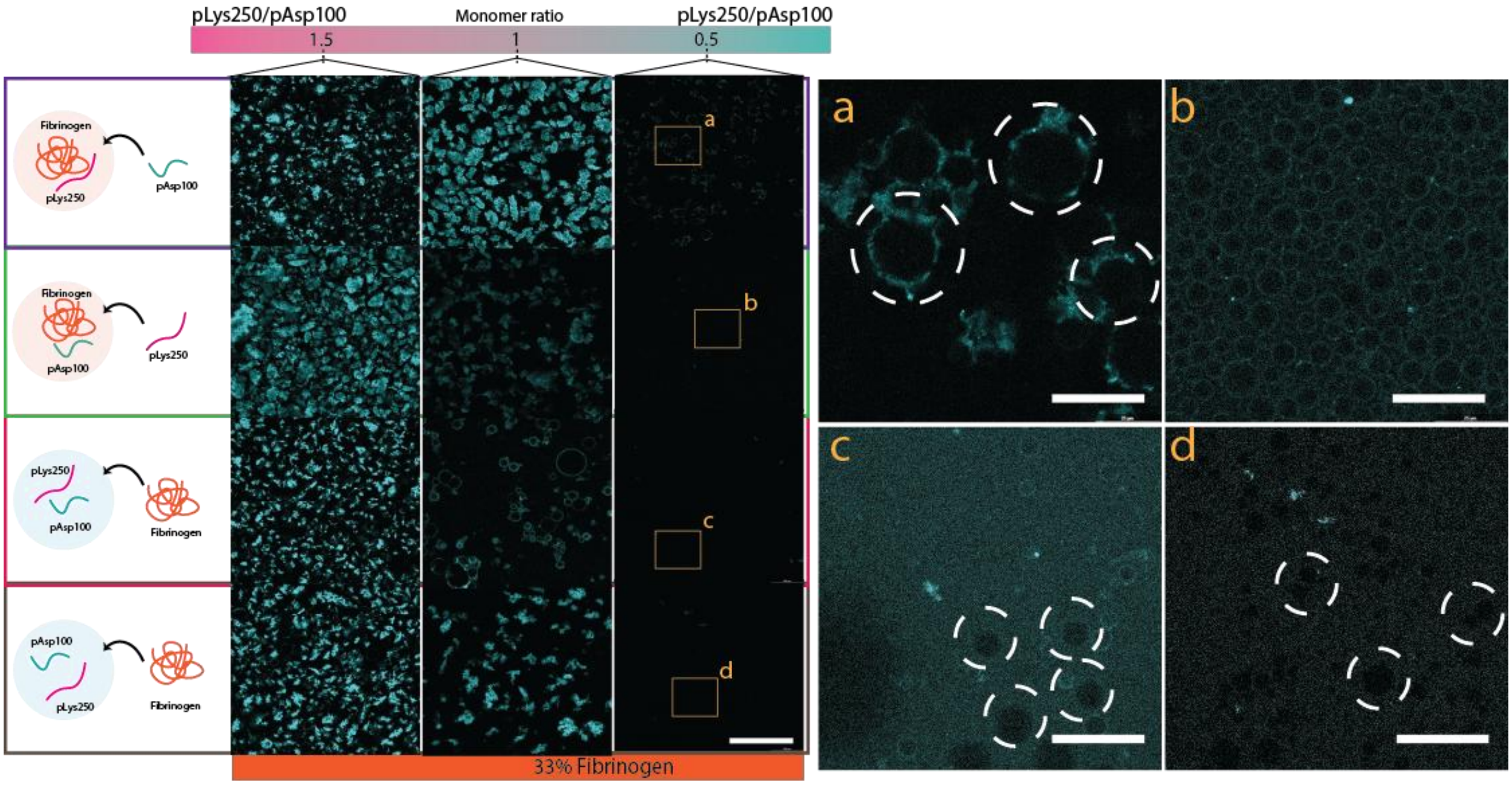

Figure S8. *The order of addition and the monomer ratio have a significant effect on the intermediary complex formation, and fibrinogen's role in the LLPS. Micrographs labelled a-d show the zoomed-in cut-outs from CLSM micrographs of 0.5 pLys250-pAsp100 monomer ratio samples. Dashed lines circle the coacervate droplets (Fibrinogen was labelled with Cy5; scale bars: 25 µm).*

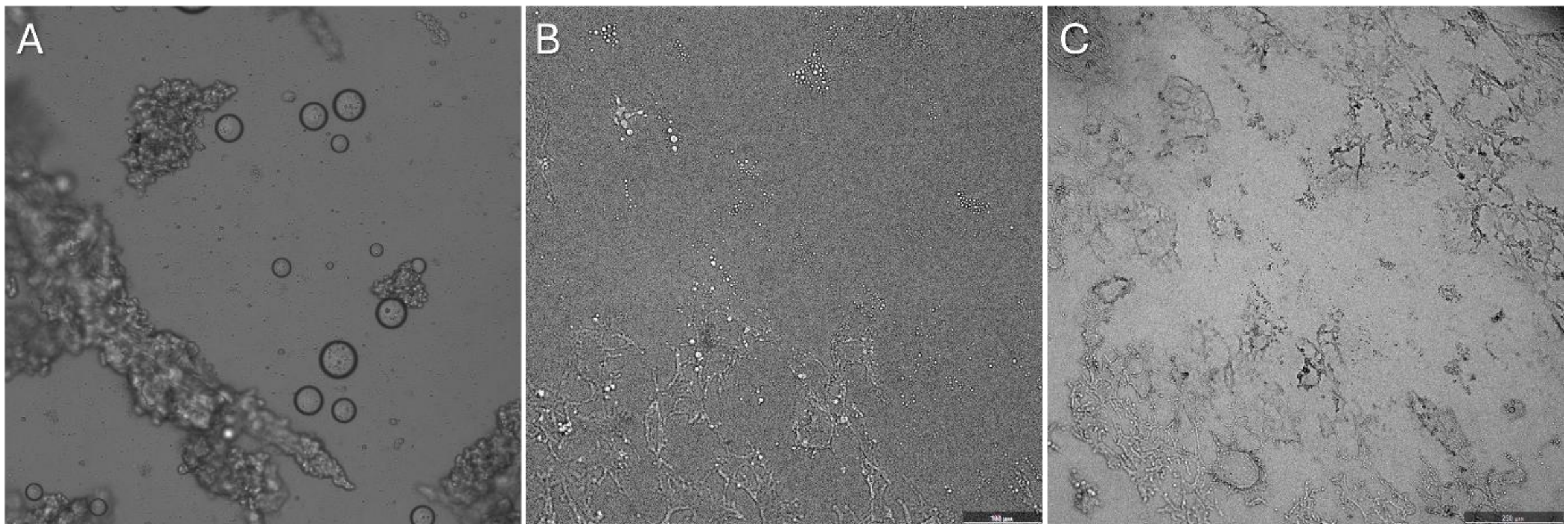

Figure S9. *The rapid drying upon LIFT printing causes structural disruption of coacervates in the printed biomaterial ink when it is not crosslinked.* ***(A)*** *Coacervates are structurally stable in hyaluronic acid biomaterial ink.* ***(B, C)*** *The stability of coacervates varied across different LIFT droplets within the same sample.*

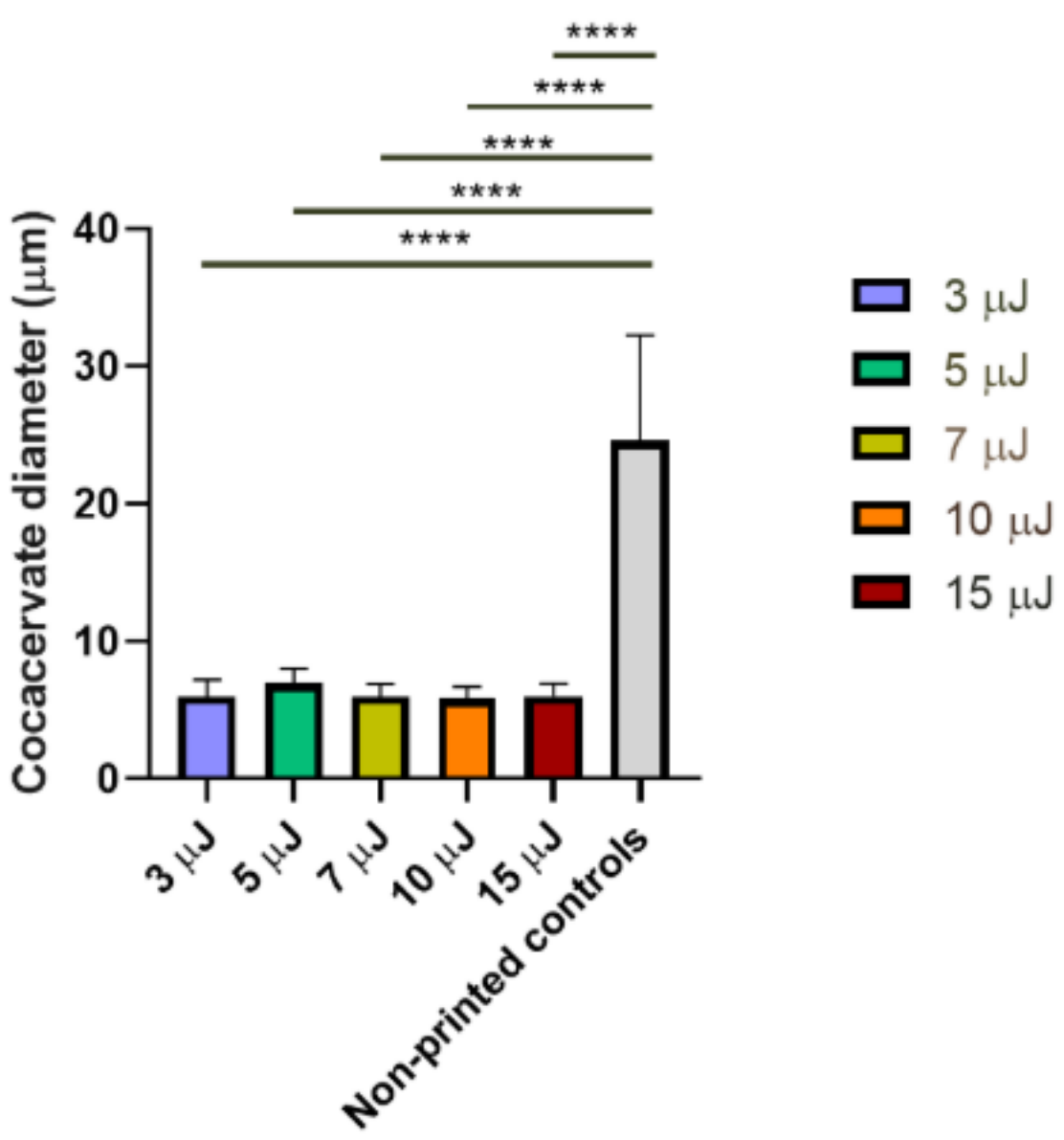

Figure S10. *Morphology comparison on non-printed and LIFT printed coacervates at laser energies 3, 5, 7, 10 and 15 µJ at constant DDR of 1.5 mm and application volume of 7 µL.*

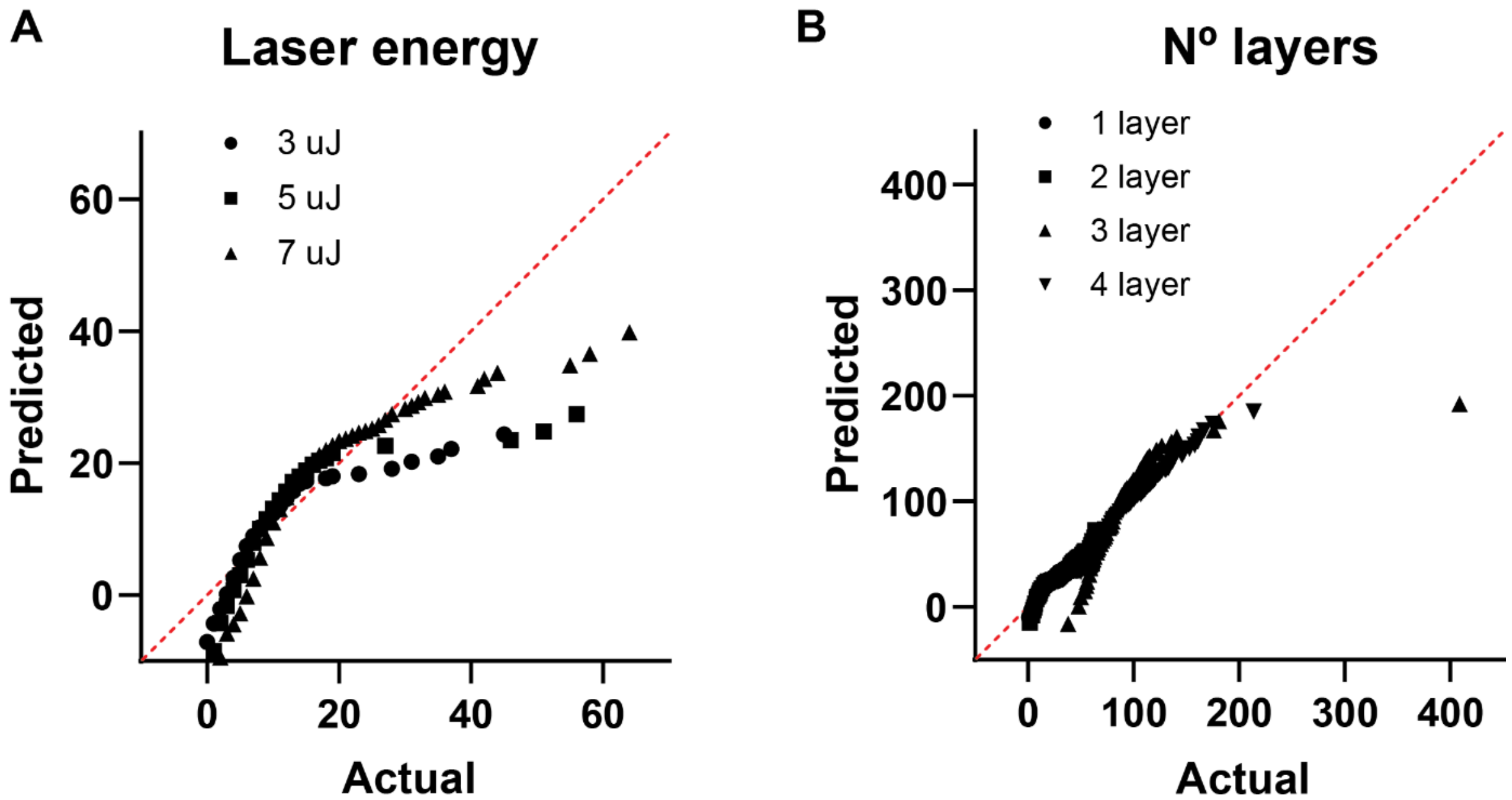

Figure S101. *Normal distribution coacervate frequency per LIFT printed droplet. A) On laser energy screening B) On number of stacked layers screening.*

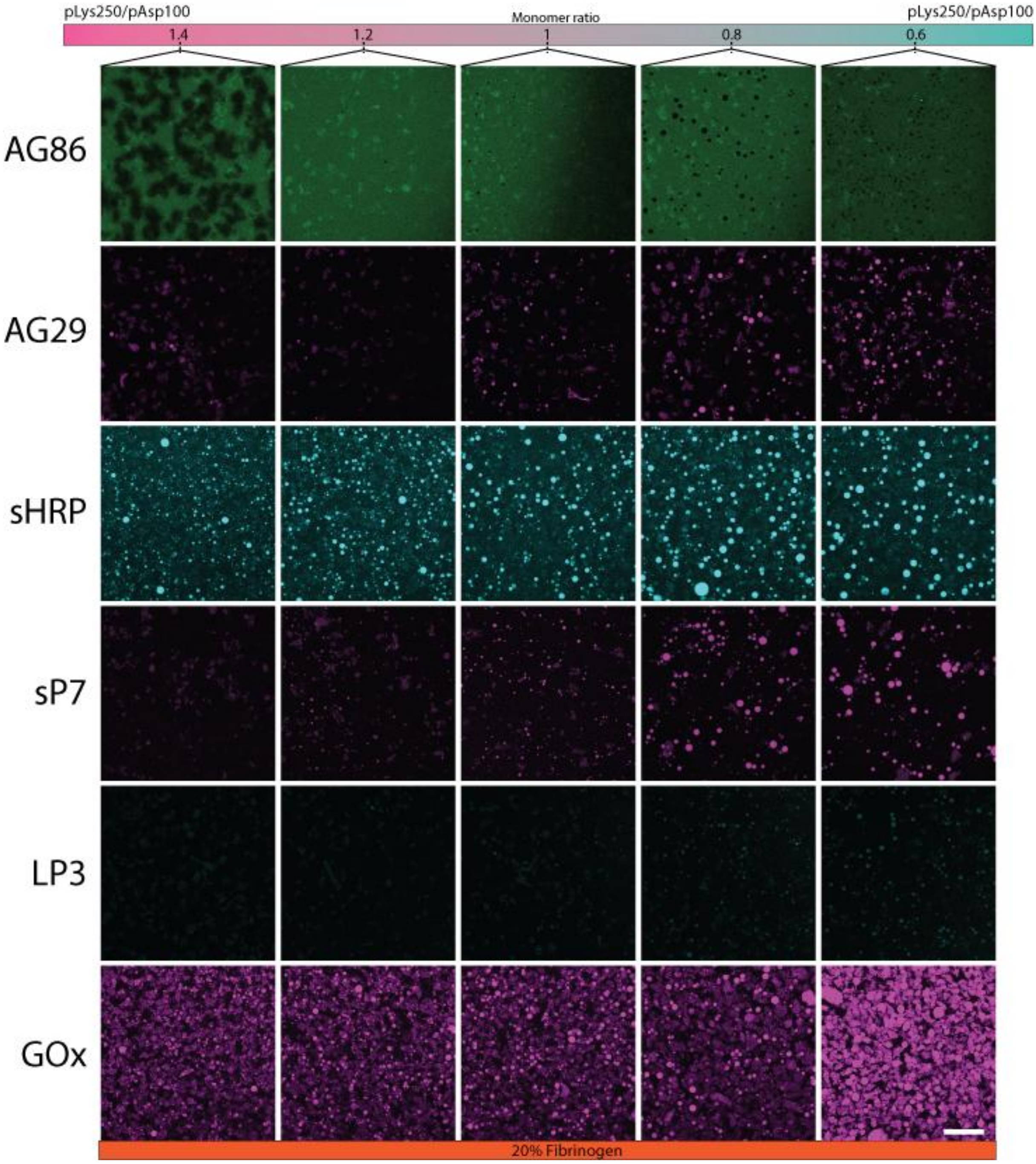

Figure S12. *Variations in the monomer ratio affect the cargo sequestration behavior of adhered complex-coacervates. AG86 and sP7 are small bit variants, and AG29 and LP3 are the large bit variants of the nano luciferase enzyme assays. The isoelectric point of the cargo molecules determines cargo sequestration behavior despite changes in molecular weight. (Scale bar: 50 µm)*

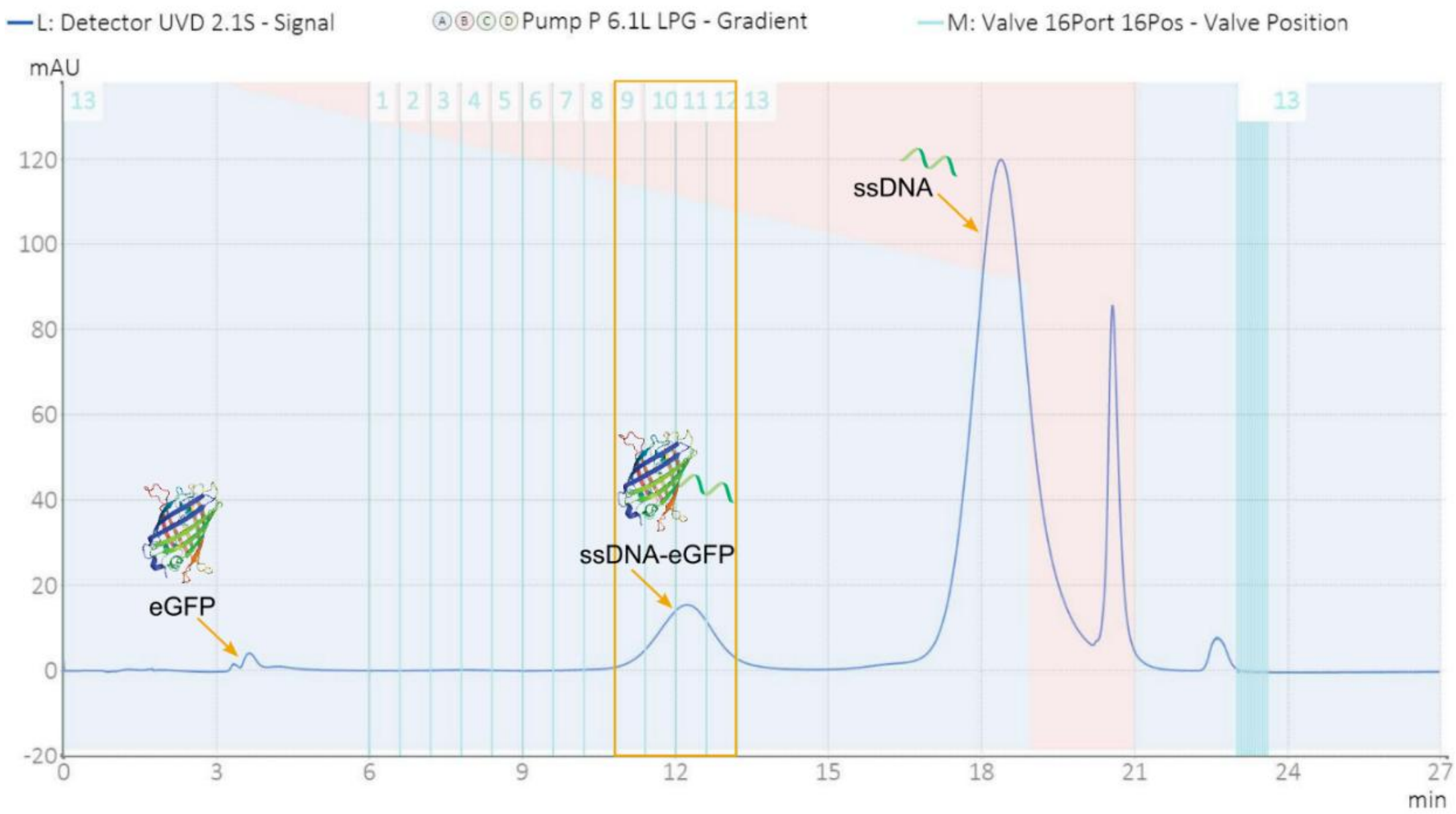

Figure S1311 *Chromatogram resulting from the anion exchange purification of the eGFP-DNA conjugation reaction mixture on the proFIRE system. Shaded regions indicate the gradient of the low- (blue) and high-salt (red) buffers. Intensity peaks correspond to non-functionalized eGFP (t=4 min), eGFP-DNA conjugate (t=12 min), and free ssDNA (t=18 min). Fractions 9-12 containing the eGFP-DNA conjugate were collected, pooled, concentrated and buffer exchanged before further usage.*

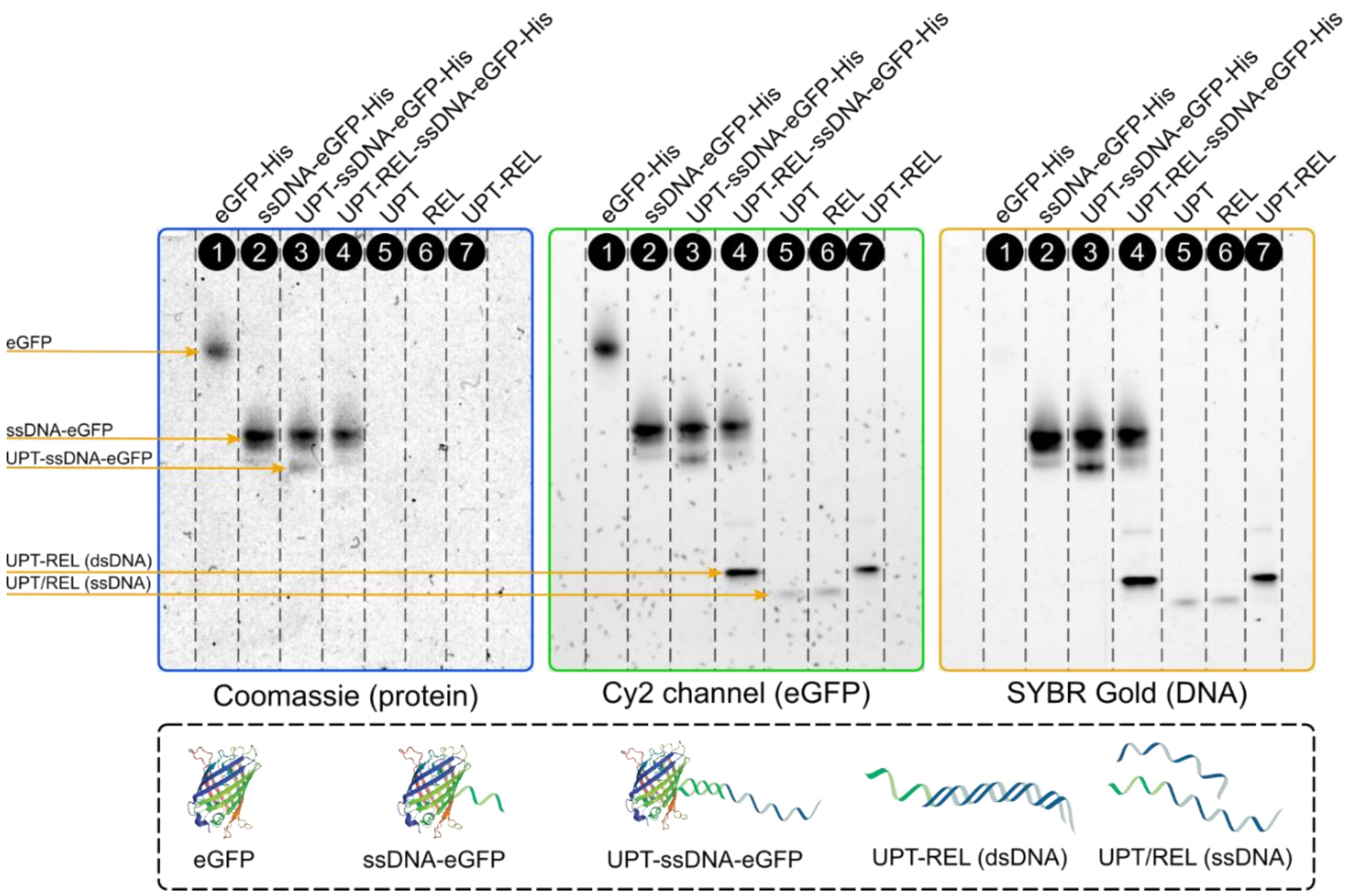

Figure S14. *Native-PAGE gel after Coomassie staining, eGFP fluorescence and SYBR Gold staining, respectively. The band in the first lane (eGFP-His; lane 1) migrates less far into the gel compared to the second lane (ssDNA-eGFP-His; lane 2). This is a result of the increase in net negative charge of the complex due to the conjugated DNA strand, causing the protein-DNA conjugate*

to migrate further into the gel towards the positive (+) electrode compared to its native state. Addition of a small quantity of the ssDNA uptake strand (UPT) leads to a further increase in net negative charge of the complex resulting in a (partial) pull-down of the protein-DNA conjugate (lane 3). Upon addition of an equal amount of ssDNA release strand (REL), the pulled-down protein fraction disappears and the protein-DNA conjugate returns to its original height, accompanied by the DNA duplex of the hybridized UPT-REL strand lower in the gel (lane 4). This was confirmed by the controls of the ssDNA UPT (lane 5) and REL strands (lane 6), and the duplex of both (lane 7).

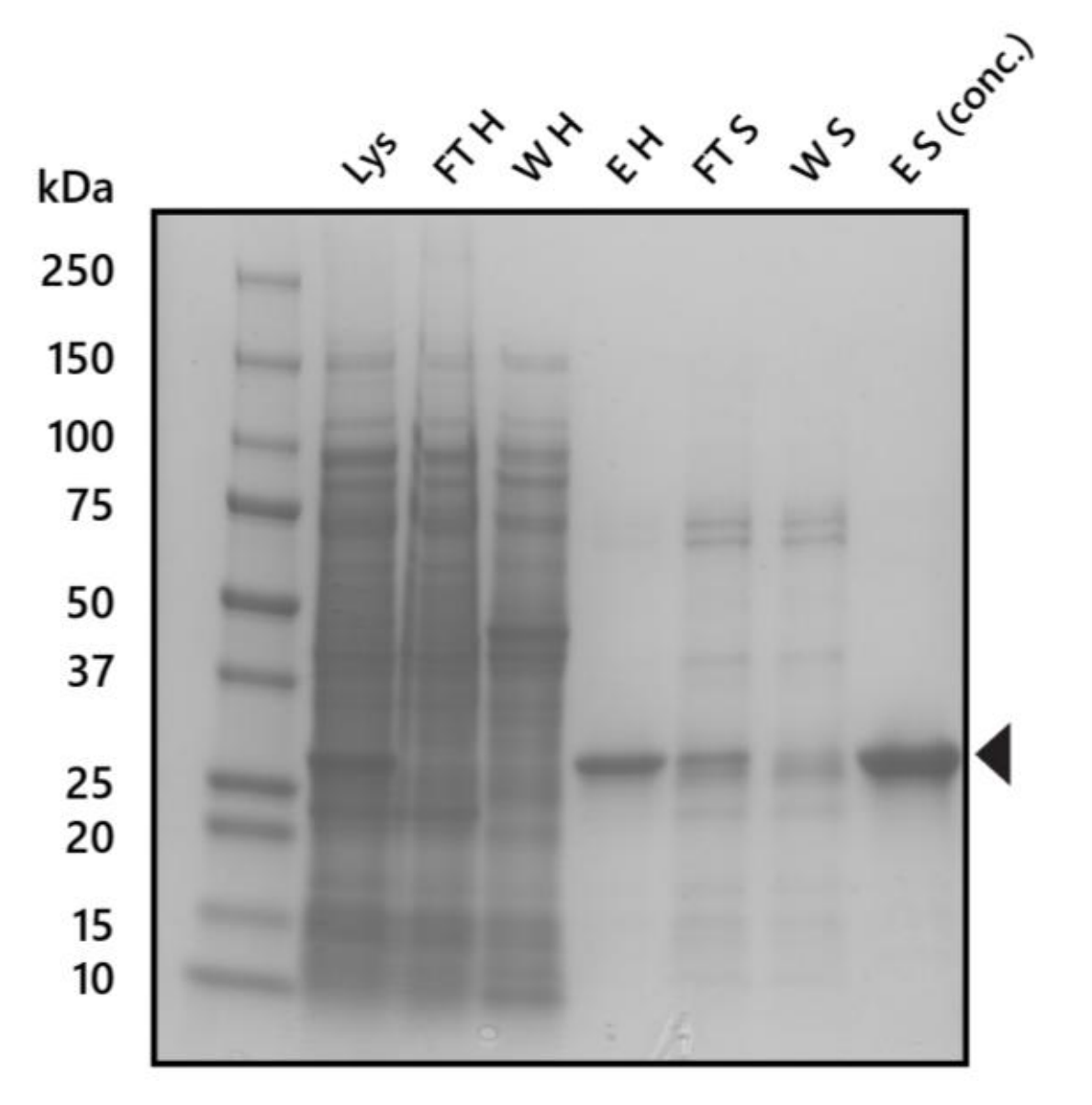

Figure S15. *SDS-PAGE analysis of eGFP_Y151pAzF following purification via Ni^{2+} NTA and StrepTactin-affinity chromatography. Anticipated mass: 30.6 kDa. Lys: Lysate, FT H, W G, E H: Flowthrough, wash and elution of Ni^{2+} NTA column. FT S, W S, E S (conc.): Flowthrough, wash and concentrated elution of StrepTactin column.*

Table S1. *Physicochemical properties of tested cargo proteins*

Cargo Protein	Molecular Weight (kDa)	pI
AG86-sBit99	30.8	8.91
AG29-dogcatch LBit	31.3	5.02
sHRP	44	<4
sBitP7	45.6	<7
LBitP3	64	<<7
GOx	160	4.2

Table S2. *Sequence and physicochemical properties of eGFP (Y151pAzF). The site for incorporation of p-azido-L-phenylalanine is indicated with an* ***X***. *Physicochemical properties were determined using the ExPASy ProtParam online tool. The extinction coefficient is provided assuming all Cys residues are reduced.*

Protein	Sequence	pI	MW (kDa)	ε (M^{-1} cm^{-1}) at 280 nm
His-eGFP_Y151X_Strep	MGSSHHHHHHSSGLVPRGSHMASMVSKGEELFTGVVPILV<br>ELDGDVNGHKFSVSGEGEGDATYGKLTLKFICTTGKLPVPWP<br>TLVTTLTYGVQCFSRYPDHMKQHDFFKSAMPEGYVQERTIFF<br>KDDGNYKTRAEVKFEGDTLVNRIELKGIDFKEDGNILGHKLEY<br>NYNSHNV**X**IMADKQKNGIKVNFKIRHNIEDGSVQLADHYQ<br>QNTPIGDGPVLLPDNHYLSTQSALSKDPNEKRDHMVLLEFV<br>TAAGITLGMDELYKSGWSHPQFEK	6.17	30.6	27390
